# Low-Temperature Synthesis and Postsynthetic Size-Tunability of AgSbS_2_ Nanocrystals and Their Application in Planar Solar Cells

**DOI:** 10.1021/acsaem.5c03034

**Published:** 2026-01-05

**Authors:** Alina Senina, Anatol Prudnikau, Angelika Wrzesińska-Lashkova, Julius Brunner, Xuan Qi, Vladimir V. Shilovskikh, Yana Vaynzof, Tilo Lübken, Fabian Paulus

**Affiliations:** † 28394 Leibniz Institute for Solid State and Materials Research (IFW) Dresden, Helmholtzstraße 20, Dresden 01069, Germany; ‡ Chair for Emerging Electronic Technologies, Technical University of Dresden, Nöthnitzer Str. 61, Dresden 01187, Germany; § Chair of Organic Chemistry I, Technische Universität Dresden, Bergstraße 66, Dresden 01069, Germany; ∥ Center for Advancing Electronics Dresden (cfaed), Technische Universität Dresden, Helmholtzstraße 18, Dresden 01069, Germany

**Keywords:** nanocrystals, cation exchange, surface chemistry, silver antimony sulfide, photovoltaics, size tuning

## Abstract

Silver antimony sulfide (AgSbS_2_) has recently emerged as a promising semiconducting material for application in optoelectronics. Its nontoxicity, earth-abundant composition, high absorption coefficient, phase, and environmental stability make it particularly interesting for photovoltaic applications. This study presents a facile room-temperature synthesis of colloidal AgSbS_2_ nanocrystals via cation exchange. The obtained AgSbS_2_ nanocrystals show a unique postsynthetic size tunability with tunable optoelectronic properties while maintaining an ideal elemental composition. The great potential of these ternary nanocrystals is demonstrated by their integration into planar nanocrystal solar cells for the first time, in which the AgSbS_2_ nanocrystals form a compact layer, reaching a promising power conversion efficiency of up to 1.99%, approaching the performance of bulk AgSbS_2_ photovoltaic cells.

## Graphical abstract

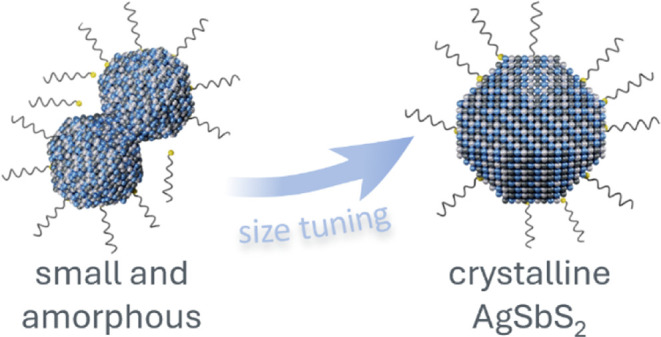

## Introduction

Colloidal nanocrystals have attracted significant research interest due to their unique physical, optical, and optoelectronic properties. The plethora of material compositions and size-dependent quantum confinement effects enable a broad tunability of the nanocrystals’ bandgap. Especially, semiconducting chalcogenide-based nanocrystals exhibit generally high stability and suitable bandgaps for various optoelectronic applications, including light-emitting diodes, catalysis, light detection, and photovoltaics.^1–5^ Among the most efficient and widely used materials, cadmium sulfide and cadmium selenide (CdS, CdSe) exhibit distinct emission peaks and high quantum yield and are suitable materials for light-emitting diodes,^6,7^ bioimaging,^8,9^ and photocatalysis.^10,11^ Lead sulfide (PbS) and lead selenide (PbSe) are well-known light-harvesting absorbers in nanocrystal solar cells with power conversion efficiencies (PCE) over 15% and 11%, respectively.^12,13^ Nevertheless, despite the remarkable advancements in chalcogenide-based nanomaterials, the cadmium (Cd)- and lead (Pb)-based semiconductors present substantial environmental concerns due to the presence of toxic heavy metals. This has led to developing and investigating new, more eco-friendly, and less toxic sustainable alternatives.^14–16^ Chalcogenide-based nanocrystal compositions based on less-toxic elements range from binary to quaternary material compositions.^17–21^ Among the binary systems, zinc sulfide (ZnS) and copper sulfide (CuS) have garnered extensive research attention due to their advantageous properties, such as high stability and ease of synthesis. ZnS quantum dots, for instance, are recognized for their exceptional optical properties and minimal toxicity, rendering them well-suited for bioimaging^22,23^ and sensor applications.^24,25^ Similarly, CuS nanoparticles have demonstrated potential in photothermal therapy^26–28^ and catalysis.^29,30^ Ternary silver bismuth sulfide (AgBiS_2_) and copper indium sulfide (CuInS_2_) nanocrystals have been shown to lead to high photovoltaic performances with power conversion efficiencies (PCE) of >10%^31^ and 8%,^32^ respectively. Despite the pioneering report of AgBiS_2_ application in solar cells in 2013 reaching a PCE of only 0.53%,^33^ the currently high performance for AgBiS_2_ was made possible by utilizing advanced surface passivation strategies,^34^ new synthetic approaches,^35^ and modified extraction layers.^36^ In the case of CuInS_2_, Wang et al. demonstrated that precise control of the Cu-to-In ratio leads to a substantial enhancement in the performance of solar cells.^32^

In addition to the extensively studied ternary compounds CuInS_2_ and AgBiS_2_, silver antimony sulfide (AgSbS_2_) has emerged as a photoabsorber and has been the focus of several studies in recent years. Like AgBiS_2_, the semiconductor has a cubic phase,^37^ a relatively narrow band gap,^37^ and a high absorption coefficient.^38^ The material has been studied for photovoltaics and photodetectors in bulk and nanocrystal form. Capistrán-Martínez et al. deposited bulk AgSbS_2_ films from solution and investigated their photoconductive properties and first solar cell performance.^39^ The films required annealing temperatures between 150 °C–280 °C to obtain AgSbS_2_ in a crystalline form. The relatively rough films exhibited a polycrystalline structure with very small domain sizes. Chalapathi et al. utilized a two-stage process for fabricating compact AgSbS_2_ layers by evaporation of Ag and Sb-precursors and sulfurization at temperatures between 300 and 400 °C.^40^ Integration of these films in planar solar cells, using CdS and Mo as charge-extraction layers, resulted in a PCE of 1.1% when processed at 350 °C. Films processed at temperatures as low as 300 °C contained Ag_3_SbS_3_ and Sb_2_S_3_ as impurities. Bai et al. studied compact and porous AgSbS_2_ layers in photovoltaic and photodetector devices on compact titania (c-TiO_2_) reaching a PCE of 1.6% with 2,2′,7,7′-tetrakis­(*N*,*N*-di-4-methoxyphenylamine4-methoxyphenylamine)-9,9′-spirobifluorene (spiro-OMeTAD) as hole extraction layer for the best compact AgSbS_2_ layer annealed at 350 °C in inert atmospheres.^41^ The pyrolysis-formation of AgSbS_2_ films at 350 °C from silver and antimony butyldithiocarbamates was studied by Lv et al., also using c-TiO_2_ and spiro-OMeTAD as extraction layers for the photovoltaic devices, resulting in a PCE of 2.09%.^42^ However, these results could only be obtained by repeating the deposition of the precursors and subsequent pyrolysis three times to obtain compact films of 150 nm thickness. The record PCE of 2.25% for compact AgSbS_2_ layers annealed at 350 °C was achieved by Zhang et al., studying the temperature-dependent annealing of spin-coated Ag–Sb-thiourea solution.^43^ While these studies on bulk AgSbS_2_ demonstrate the potential of this material, the high annealing temperatures render this approach incompatible with flexible substrates. Low-temperature processing of AgSbS_2_ in the form of crystalline and high-quality nanocrystal inks could circumvent this problem. However, reports on AgSbS_2_ nanocrystals or their use in optoelectronic devices are scarce. The first use of AgSbS_2_ nanocrystals with a size of ca. 40 nm in Grätzel-type solar cells has been reported by Ho et al. already in 2013 using the successive ionic layer adsorption and reaction (SILAR) method.^44^ The device performance of such AgSbS_2_-sensitized devices reached a PCE of only 0.34% for the optimized deposition of three SILAR cycles. AgSbS_2_ nanocrystals of various sizes and spherical shapes were demonstrated by Zhou et al. using reaction temperatures of 180–220 °C, resulting in relatively large, crystalline nanocrystals with a cubic phase.^37^ The synthesis occurred from a one-pot mixture that was heated while reaction temperature, duration, and sulfur source and content determined the resulting shape and size of the obtained AgSbS_2_ nanocrystals. Notably, the authors have not demonstrated the ability to perform ligand exchange on their AgSbS_2_ nanocrystals, most likely due to the use of dodecanethiol (DDT) as a strongly coordinating ligand on the nanocrystal surface. As a consequence, synthetic approaches with DDT are incompatible with potential application in functional devices that rely on electronic coupling between the nanocrystals. A similar synthetic approach was used by Choi et al. to study the surface chemistry of AgSbS_2_ nanocrystals and their conductivity in the dark after passivation with methylammonium lead iodide.^45^ Schottky-type Al/Si photodiodes with AgSbS_2_ interlayer for light detection were fabricated by Koçyiğit et al., who utilized a hot-injection method to obtain AgSbS_2_-nanocrystals of ca. 35 nm in diameter.^46^

The small number of studies dedicated to AgSbS_2_ nanocrystals could be attributed to the difficulty in synthesizing the material using the commonly employed hot-injection method. In this method, the affinity of softer silver Ag^+^ to soft sulfur exceeds the affinity of hard antimony Sb^3+^. This behavior gives rise to the formation of silver-rich nanoparticles, a broad size distribution, and an irregular composition, which all negatively impact a potential solar cell performance. Consequently, alternative synthetic approaches providing precise composition and size control could facilitate research on AgSbS_2_ nanocrystal-based optoelectronics.

This study presents a cation exchange process for synthesizing AgSbS_2_ nanocrystals. This approach entailed the synthesis of small Ag_2_S nanocrystals under ambient conditions and room temperature using bis­(lauroyl) sulfide as a new and air-stable sulfur precursor. Subsequently, a rapid cation exchange of Ag^+^ to Sb^3+^ was executed using antimony-(III)-chloride (SbCl_3_) and the soft Lewis base trioctylphosphine (TOP) at room temperature. The cation exchange process was found to be rapid, resulting in an immediate change in the absorption edge and a phase change. After purification, the resulting AgSbS_2_ nanocrystals exhibited a unique postsynthetic size-tunability, and annealing of the nanocrystal solution at 150 °C allows for tuning the nanocrystal size up to an average size of 9.1 nm while also improving their crystalline ordering. Integration of such AgSbS_2_ nanocrystals in planar solar cells resulted in a photovoltaic performance of up to 1.99% PCE, the highest reported performance for AgSbS_2_ nanocrystals and remarkably close to the record performance of 2.25% PCE reported for bulk AgSbS_2_ layers fabricated at high temperatures.

## Results and Discussion

Ag_2_S nanocrystals were synthesized at room temperature by rapidly injecting a new sulfur precursor, bis­(lauroyl) sulfide (La_2_S), into a silver nitrate (AgNO_3_)/oleylamine (OAm) solution (molecular structure, and analytical data for La_2_S see Figures S1 and S2). Similarly to bis­(stearoyl) sulfide (St_2_S), which we have recently reported for the synthesis of metal chalcogenides, such as PbS or AgBiS_2_,^47,48^ La_2_S is a colorless solid that is stable to moisture and air and can be readily employed in nanocrystal synthesis. However, a notable advantage of La_2_S over St_2_S is its higher solubility in toluene at room temperature making it more favorable for reactions at ambient conditions. The synthesized and purified Ag_2_S nanocrystals exhibited an average size of (4.4 ± 0.6) nm, slightly larger at the same reaction conditions than St_2_S due to differences in the precursor reactivity (Figure S3). The Ag_2_S nanocrystals serve as a template for the following cation exchange reaction with Sb^3+^ to obtain AgSbS_2_ nanocrystals (Figure 1a), adopting our previously published approach for the synthesis of AgBiS_2_.^48^ The driving force for this cation exchange is determined by the choice of ligand and the size of the cations. Trioctylphosphine, a soft base widely used in cation exchange reactions and capable of strong coordination with soft acids through π-bonding interactions, was used as a ligand, resulting in the efficient removal of Ag^+^ cations from the nanocrystal lattice.^49,50^ The cation exchange reaction with Sb^3+^ proceeds almost instantaneously, within 5 min, considerably faster than the analogous reaction with Bi^3+^ to AgBiS_2_, which also requires slightly elevated temperatures to commence at reasonable rates.^48^ This is most likely the consequence of a smaller ionic radius of Sb^3+^ compared to Bi^3+^ and an easier diffusion and incorporation into the Ag_2_S lattice. Figure 1b illustrates the transmission electron microscopy (TEM) images of Ag_2_S and AgSbS_2_ nanocrystals after cation exchange. The initial average nanocrystal size of Ag_2_S (4.4 ± 0.6) nm is maintained with an average size of (4.1 ± 0.5) nm upon the introduction of antimony (Figure S4). As seen from Figure 1b, AgSbS_2_ nanocrystals appear less spatially separated after cation exchange and tend to attach closer to each other, which could be indicative of modified surface ligands and will be further discussed below. The AgSbS_2_ nanocrystal dispersions, however, maintain their colloidal stability for long periods of time and do not form aggregates or precipitates.

**Figure 1:**
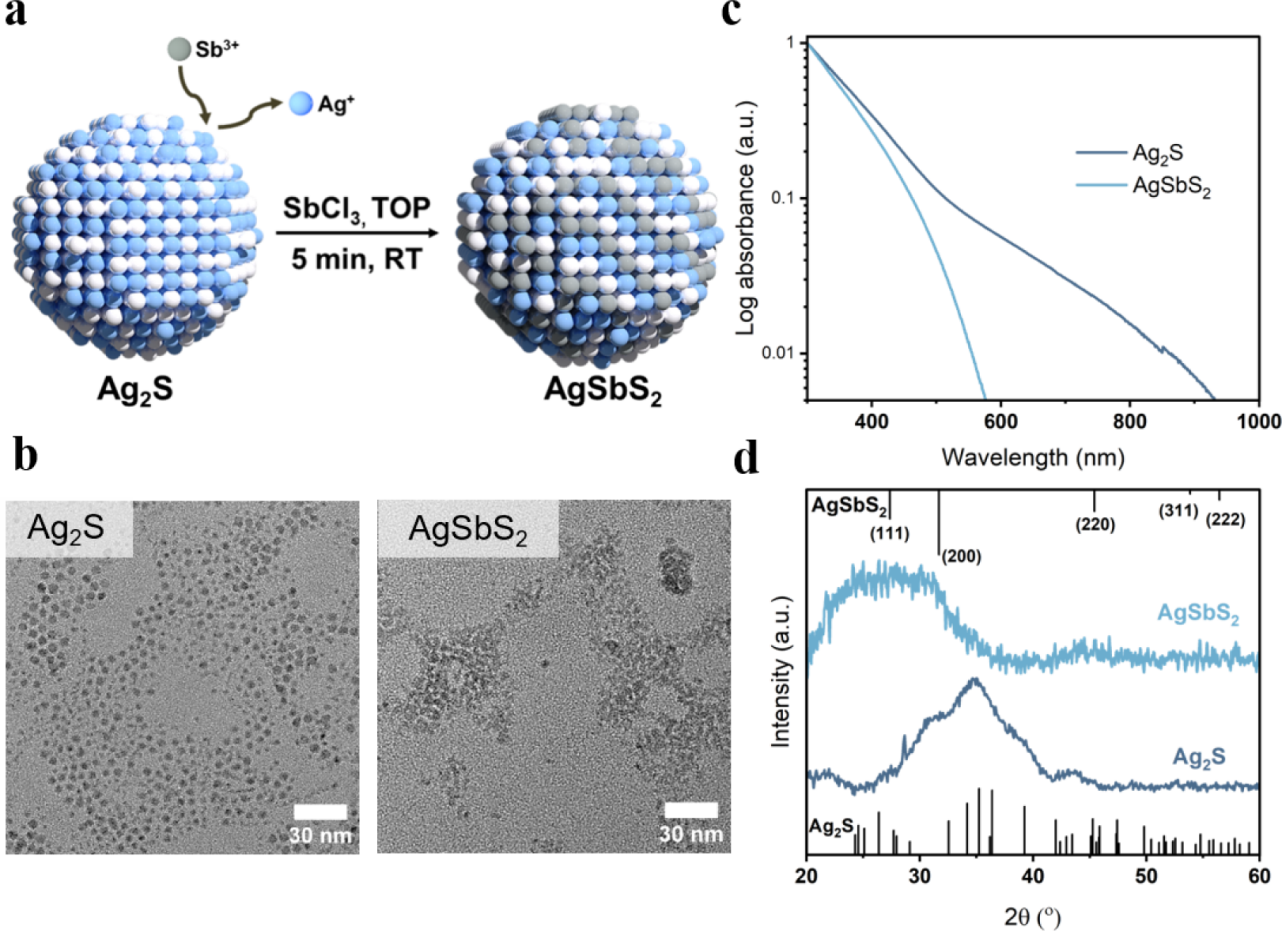
(a) Schematic representation of AgSbS_2_ cation exchange synthesis at room temperature. (b) TEM images of Ag_2_S and AgSbS_2_ nanocrystals synthesized at RT. (c–d) Absorbance spectra and XRD pattern of Ag_2_S and AgSbS_2_ NCs (reference pattern obtained from ref 51 for Ag_2_S (CCDC 1692248) and ref 52 for AgSbS_2_ (CCDC 1613740).

Figure 1c depicts the absorption spectra of Ag_2_S and AgSbS_2_ nanocrystals in solution after cation exchange. It is evident that following cation exchange, there is a notable blue shift in the absorption edge toward 600 nm. This shift is also correlated with the rapid change in the color of the nanocrystal dispersion from dark brown to bright orange immediately following the injection of antimony (Figure S5). These observations indicate a change in the electronic structure of the nanocrystals and agree well with the expected change in the band gap for the two materials. Silver sulfide exhibits a bandgap of ca. 0.9 eV for bulk Ag_2_S,^53^ while the bandgap for poorly crystalline to amorphous AgSbS_2_ has been reported to be approximately 1.7 eV.^54^ In addition, a transition in the X-ray diffraction (XRD) pattern is observed, as evidenced by Figure 1d. The broad diffraction features centered around 34.7° attributed to Ag_2_S nanocrystals in the monoclinic β-Ag_2_S phase vanish, and a broad reflection around 27.6° emerges, which agrees well with the position of expected reflections for cubic AgSbS_2_. The broad and not-well-defined nature of these reflections is attributed to the small nanocrystal size of ca. 4 nm and could reflect poor long-range ordering within each nanocrystal. We believe that the latter effect might dominate here because a crystalline, well-ordered ternary AgSbS_2_ lattice has been widely observed in literature only for high annealing temperatures compared to our cation exchange reaction conducted at room temperature. Despite the cubic phase, the AgSbS_2_-nanocrystals appear irregular and more spherical.

To improve the lattice ordering and crystallinity, we annealed the purified AgSbS_2_ nanocrystals in solution at 150 °C for short periods of time. Upon thermal treatment, the optical absorbance spectra shifted toward longer wavelengths (Figure 2a), causing the nanocrystal dispersion to change color and darken significantly (Figure S6), which could indicate changes to the bandgap of the nanocrystals, which is discussed later. In addition, the XRD patterns improved through the thermal treatment, resulting in the appearance of the corresponding reflections of cubic AgSbS_2_ after 60 s and a profound sharpening for 90 and 120 s, suggesting not only the intended improvement in lattice ordering but also an increase in nanocrystal size (Figure 2b). These findings were further confirmed by TEM, revealing a monotone increase in nanocrystal sizes with the duration of the thermal treatment (Figure 2c). Size analysis showed that the nanocrystal size increases from the initial AgSbS_2_-nanocrystals obtained and purified after the cation exchange at room temperature (RT) from (4.1 ± 0.5) nm to (4.4 ± 0.7) nm after 30 s, (5.8 ± 0.6) nm after 60 s, (6.6 ± 1.1) nm after 90 s, and finally (9.1 ± 0.9) nm after 120 s while all nanocrystal inks remain their colloidal stability (Figure S7). The larger nanocrystals obtained by the thermal annealing in solution exhibit significantly improved lattice ordering as is indicated by the XRD patterns and directly visualized in high-resolution TEM (Figure S8). The nanocrystals exhibit well-defined crystal facets, but the overall shape remains irregular and closer to a spherical morphology. Longer annealing times at 150 °C further increase the nanocrystal size but result in a loss of colloidal stability and, finally, in precipitation of AgSbS_2_ aggregates. The observed postsynthetic and postpurification size-tunability is unusual and must originate from a modified nanocrystal surface chemistry. Yet, it provides an additional handle to tune the optoelectronic properties of semiconducting nanocrystals for optoelectronic applications. We highlight that such a postsynthetic size-tunability was not observed in our previous study on the cation exchange to form AgBiS_2_ nanocrystals and seems to be a unique characteristic of the AgSbS_2_-nanocrystals. To investigate the origin of this behavior further, we studied the AgSbS_2_-surface chemistry in more detail. A fusion and growth of AgSbS_2_ upon thermal treatment suggest the presence of nonpassivated nanocrystal facets or their exposure in solution upon ligand-dissociation, allowing the fusion and coalescence of small nanocrystals into larger ones.

**Figure 2:**
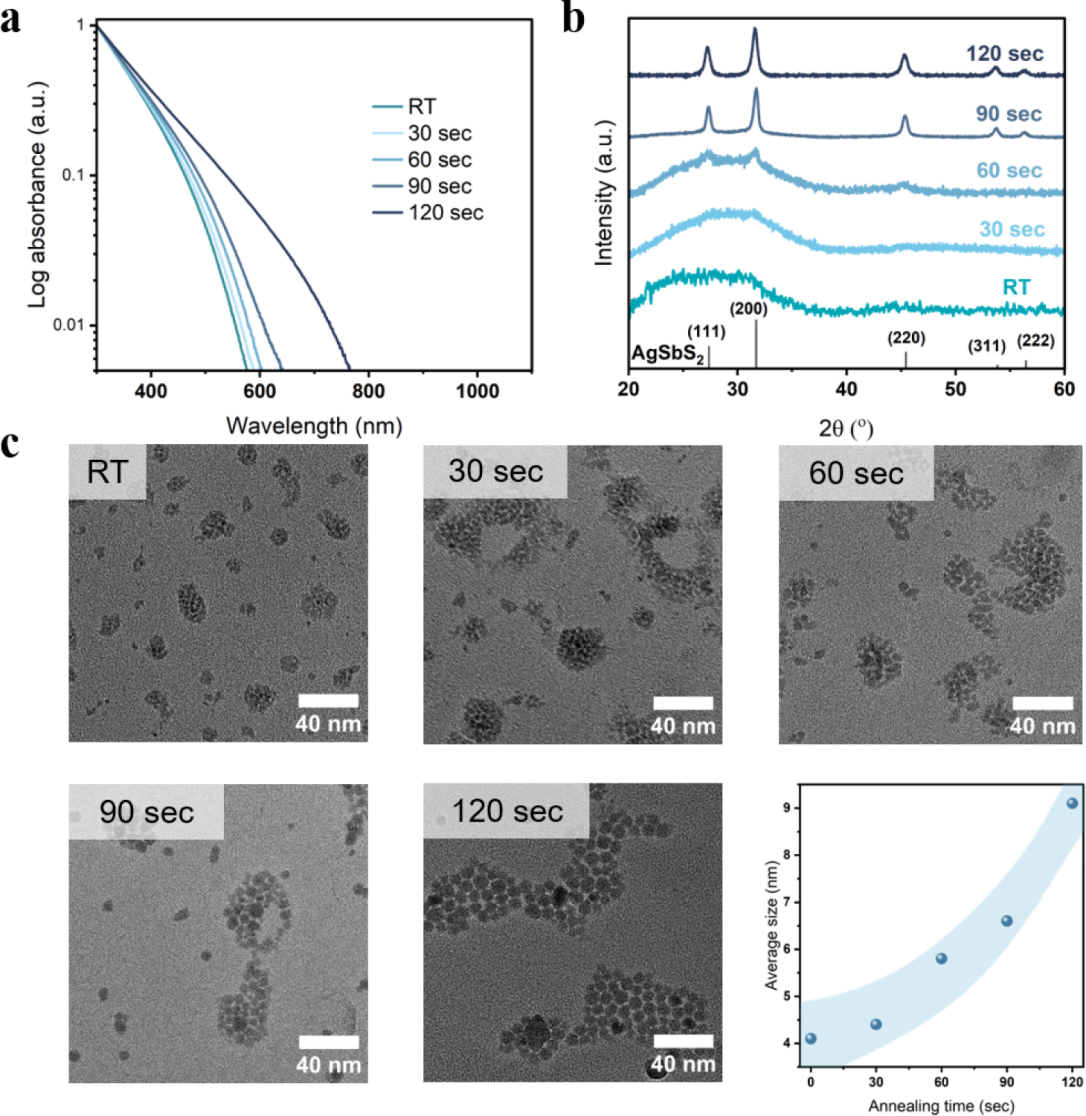
(a) Optical absorbance spectra of AgSbS_2_ nanocrystals in solution after different thermal annealing times at 150 °C. (b) XRD pattern of AgSbS_2_ NCs at different annealing times, and (c) corresponding TEM images of the nanocrystals. RT = as synthesized at room temperature.

Figure 3 offers a schematic of the ligand dissociation and AgSbS_2_ nanocrystal coalescence process occurring during thermal annealing in solution. In a diluted and purified solution, when the overall concentration of free ligands is low, the absorption–desorption equilibrium for dynamic surface ligands tend to shift toward the direction of desorption (Figure 3a). With increasing temperature, the equilibrium shifts further toward desorption, resulting in a ligand deficiency on the surface of nanocrystals which allows the fusion and coalescence of small nanocrystals into bigger ones (Figure 3b).

**Figure 3:**
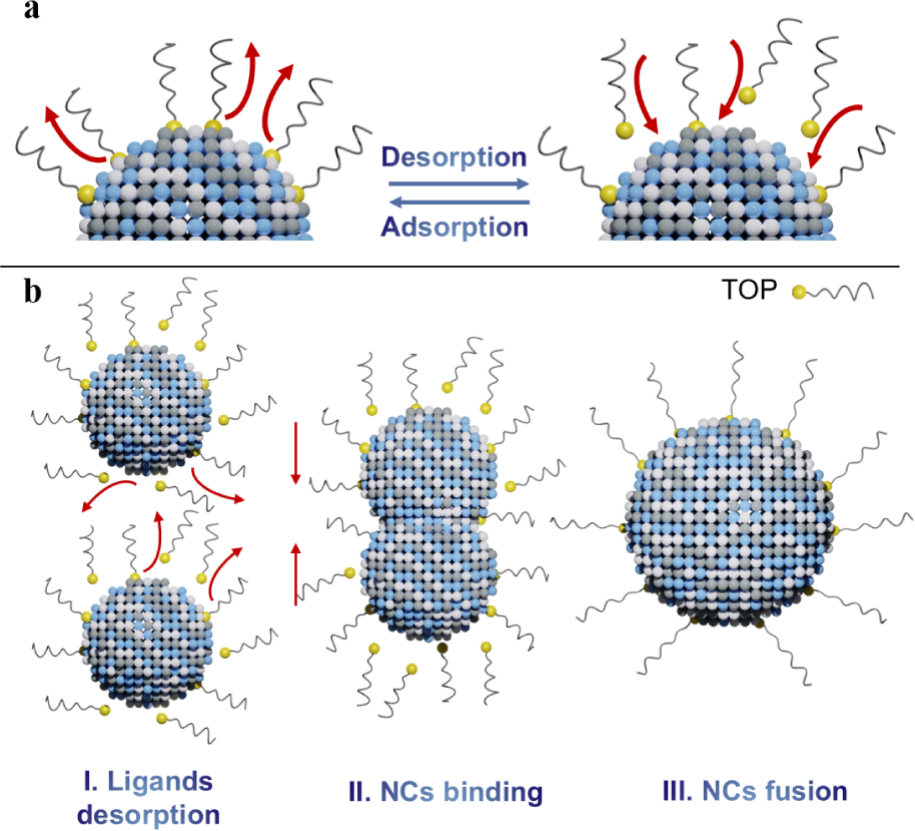
(a) schematic representation of the dynamic ligand equilibrium on the nanocrystal surface and (b) AgSbS_2_ nanocrystal fusion enabled by ligand desorption via thermal annealing of the colloidal solution.

To identify the type of ligand on the purified AgSbS_2_ nanocrystal surface, we performed nuclear magnetic resonance (NMR) measurements of the purified nanocrystals after cation exchange (Figure 4a). Interestingly, we are unable to detect any ^1^H-NMR resonances that are related to the presence of OAm although an excess of OAm was used during the synthesis, and it is typically the dominating ligand in the stabilization of Ag_2_S nanocrystals used as starting materials for our AgSbS_2_ nanocrystals (NC). Instead, our measurements reveal the presence of TOP or its oxidized product tri­(octyl)­phosphine oxide (TOPO) in the nanocrystal dispersion. Even though only TOP was added to the cationic exchange reaction, the entire process, including purification, was carried out in ambient conditions, which can lead to partial or complete oxidation of TOP, resulting in the formation of TOPO. Due to the similarities in the ^1^H-NMR spectra of TOPO and TOP, it is impossible to differentiate each species’ individual contributions in the ^1^H-NMR spectra of the AgSbS_2_-nanocrystal dispersion. The broadening and slight downfield shift of the resonances between 0.8 and 1.7 ppm are characteristic of surface-bound ligands since broadening is typically pronounced for protons closer to the nanocrystal surface.^55^ These findings suggest that the cation exchange reaction not only resulted in the replacement of Ag^+^ with Sb^3+^ in the crystal lattice but also led to a replacement of ligands from OAm to TOP/TOPO on the nanocrystal surface.

**Figure 4:**
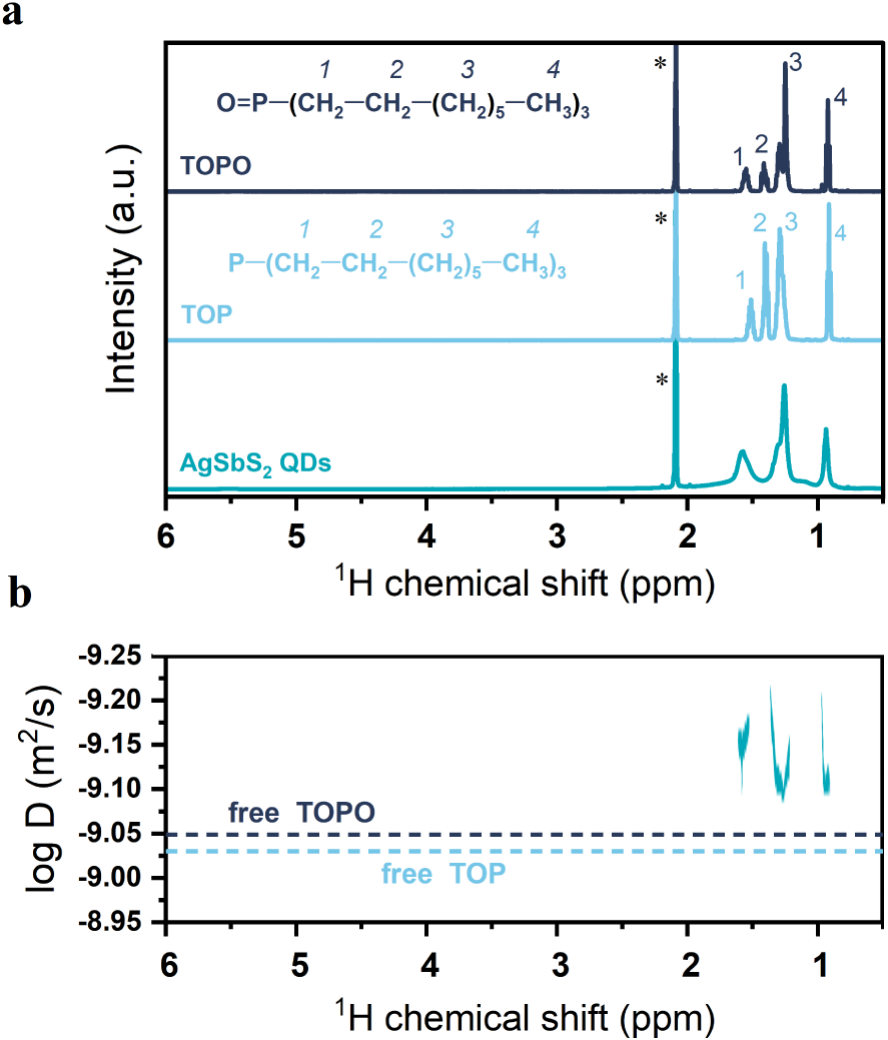
(a) ^1^H-NMR spectra of plain TOPO (top), plain TOP (middle), and AgSbS_2_ nanocrystal dispersion (bottom), all recorded in toluene-d_8_. Residual proton signals from toluene-d_8_ marked as (*). (b) ^1^H-DOSY NMR spectrum of AgSbS_2_ nanocrystals (60 mg/mL) after purification in toluene-d_8_. For comparison, the diffusion coefficients of pure TOPO and pure TOP in toluene-d_8_ are displayed as dashed lines.

To further confirm that TOP or TOPO interact with the nanocrystal surface, we conducted diffusion-ordered spectroscopy NMR measurements (^1^H-DOSY-NMR) in which the diffusions of molecules in the solution can be assessed (Figure 4b). These measurements show that the observed ^1^H-NMR resonances of TOP/TOPO exhibit a decreased diffusion coefficient compared to free TOP and TOPO molecules and must stem from molecules interacting with the nanocrystal surface (reference measurements see Figure S9). The small difference in the diffusion coefficient for surface-bound ligands and free molecules (TOP and TOPO), along with the lack of a signal from unbound ligands in the solution of NCs, suggests a rapid exchange between the free and bound states of the ligands. As shown by others, fast ligand adsorption/desorption exchange results in only one signal on the ^1^H-DOSY-NMR spectra, corresponding to the average diffusion coefficient of both bound and unbound species.^56^ This suggests that in the case of purified AgSbS_2_-nanocrystals, ligands can rapidly desorb, leaving nonpassivated nanocrystal facets behind which could be the origin of the observed nanocrystal fusion. To further confirm the change in surface ligand from OAm to TOP­(O), we employed Fourier-Transform Infrared Spectroscopy (FTIR). The measurements (Figure S10) confirm the absence of characteristic bands for OAm after the cation exchange, in line with the NMR measurements presented in Figure 4a.

To elucidate which facets are responsible for the observed fusion of nanocrystals during thermal annealing, we studied the growth of nanocrystals in the presence of different ligands (X-, Z-, and L-types). We monitored the thermally induced size increase via TEM for samples obtained for annealing times of 60 and 120 s at 150 °C. Figure 5 summarizes the nanocrystals morphology after the addition of 1% silver oleate (Z-type), 1% oleic acid (X-type) and 1% OAm. In the case of added Z- and X-type ligands, the average size of AgSbS_2_ nanocrystals changes similarly to reference AgSbS_2_ NCs after annealing and the overall growth remains unaffected. This suggest that X- and Z-type ligands do not bind strongly to the nanocrystal surface or, alternatively, fusion occurs through facets that are not well passivated by these types of ligands. However, adding 1% OAm (L-type) effectively suppresses the nanocrystal growth, while the small nanoparticles tend to form larger aggregates on the TEM grid due to the excess of OAm. These findings are supported by time-resolved optical absorbance spectroscopy in solution (Figure S12). The absorbance spectra of the purified AgSbS_2_ nanocrystals in solution undergo a continuous red shift during thermal annealing, due to improved order and growth in size. This behavior is no longer observed in the presence of 1% OAm and the nanocrystals exhibit only a minimal red shift following the thermal annealing procedure. These measurements further confirm that the nanocrystals are not dissolved upon the addition of 1% OAm. We further conducted NMR studies on AgSbS_2_ nanocrystals treated with OAm and extensive washing (see Figure S13). The ^1^H-NMR spectrum of such nanocrystals confirms the presence of bound and free OAm in contrast to the NMR spectrum after the cation exchange synthesis (see Figure 4a), in which no contribution from OAm was observed. Considering that neutral OAm, similarly to TOP/TOPO, preferentially binds to neutral nanocrystal facets, we conclude that the fusion of AgSbS_2_ nanocrystals occurs through the (100)-facets. These findings agree well with the study by Choi et al., who demonstrated that AgSbS_2_ nanocrystals contain neutral Ag-rich (100) facets that bind rapidly to thiolate and amine ligands while the charged (111) facets contains both Ag and Sb species.^45^

**Figure 5:**
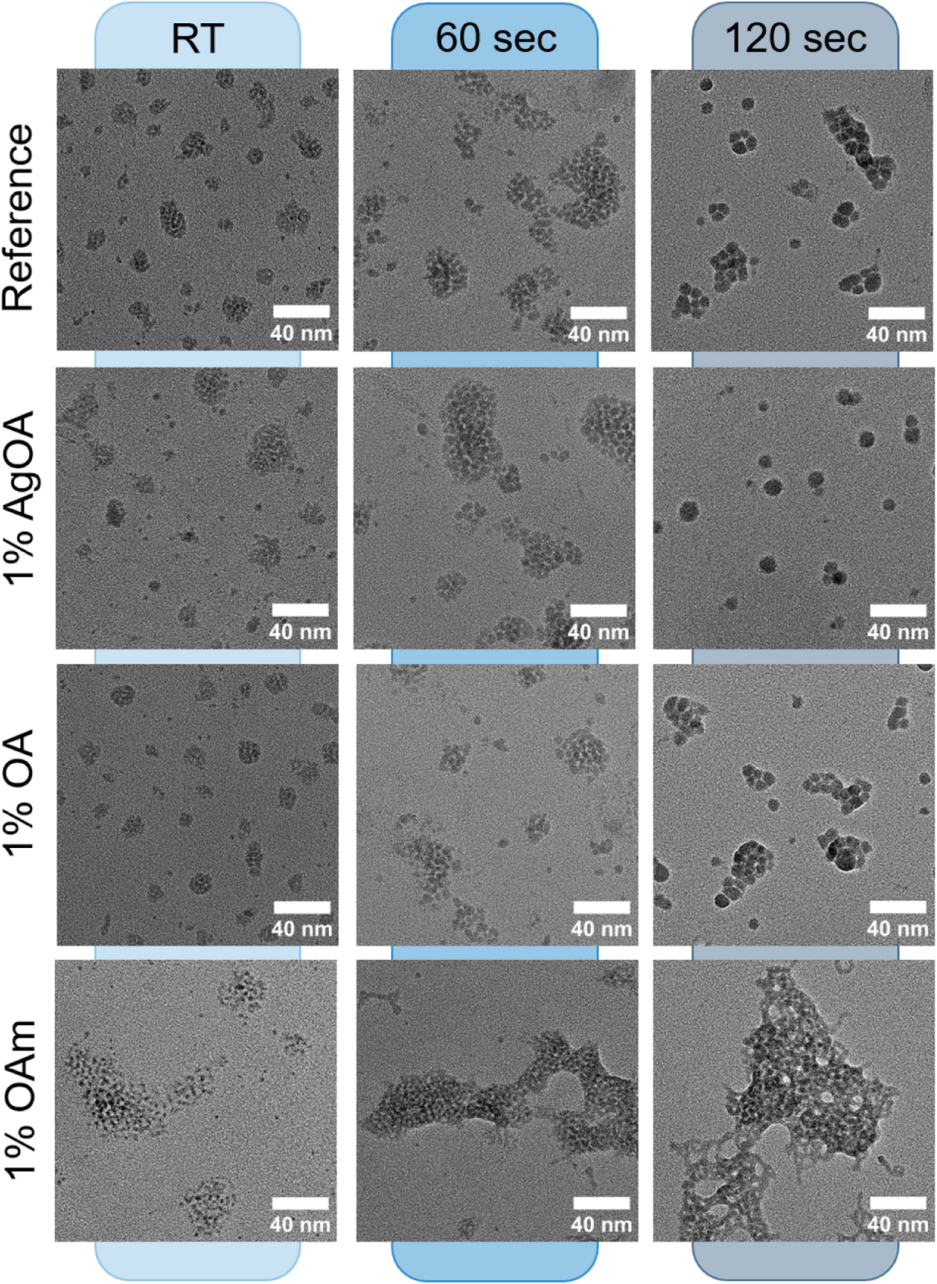
TEM images of AgSbS_2_ with the addition of Z, X, and L-type ligands at different annealing times. Corresponding NC sizes can be found in Figure S11.

The composition of the AgSbS_2_ nanocrystals obtained by cation exchange before and after the thermal annealing was probed utilizing X-ray photoemission spectroscopy (XPS). We spin-coated the nanocrystal dispersion on SnO_2_ to obtain thin films and annealed all films for 30 s at 150 °C to remove residual solvent. We compare films as deposited to those, for which we additionally performed a solid phase ligand exchange (SPLE) with 3-mercaptopropionic acid (MPA) in methanol to replace the long-chain organic ligands. All films exhibit well-defined spectra of the expected elements and show doublets affiliated with Ag 3d, Sb 3d, and S 2p (Figure 6 and Figure S14).

**Figure 6:**
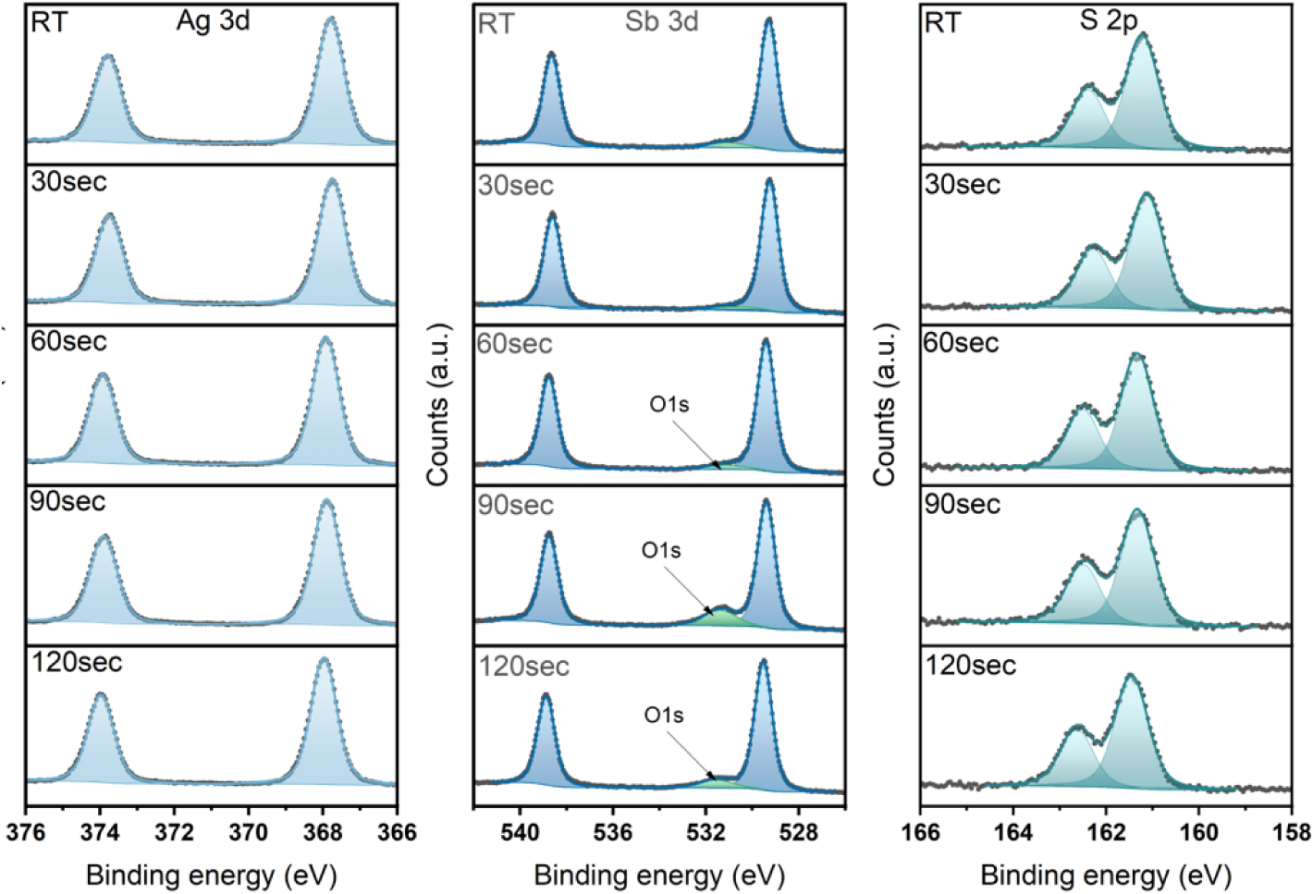
XPS spectra of Ag 3d, Sb 3d, and S 2p for spin-coated AgSbS_2_ nanocrystals on SnO_2_ treated with MPA after different annealing times in solution.

Samples without the SPLE treatment show a nonideal stoichiometric Ag:Sb ratio of ca. 3:4, indicating an excess of antimony in the films (Table S1). The Sb 3d spectra contain notable amounts of a higher binding energy species of antimony that could be attributed to SbCl_3_ and/or Sb–O. In addition, traces of chlorine can be identified. These findings point toward potential impurities and nonreacted antimony precursors that remain in the film. In addition, the films exhibit significant amounts of carbon from the long-chain organic ligands, as discussed above. Thermal annealing of the nanocrystals in solution at 150 °C does not change the overall composition of the nanocrystal film. However, in the Ag 3d scan a shoulder appears at higher binding energies that could be attributed to elemental silver pointing toward nonperfectly passivated nanocrystal surfaces or possible reactions with some of the side products.

For SPLE-treated films, the results differ significantly (Figure 6). The Sb 3d scans reveal only one species with a doublet at binding energies of 538.8 and 529.4 eV, with only a minor contribution of an oxygen singlet associated with O–H groups originating from exposure to the ambient. In addition, the silver 3d spectra exhibit clear doublets at binding energies of 373.9 and 367.9 eV, with no other silver species observed for the plain nanocrystals (RT) or all annealing times in solution. The XPS results for SPLE-treated samples confirm a slight excess of Ag over Sb, as expected based on the dominance of silver-terminated (100) facets (Table 1, Table S2). Overall, the nanocrystal composition of Ag:Sb:S was found to be 0.6:0.5:1, which is remarkably close to the stoichiometric composition of 0.5:0.5:1. The ideal composition and purity of the nanocrystals are even confirmed for AgSbS_2_ nanocrystals that did not undergo any thermal annealing in solution. This highlights the great potential of the cation exchange process that enables the synthesis of near-perfect stoichiometric AgSbS_2_ nanocrystals, unlike the hot-injection synthesis, where the differences in Ag and Sb reactivity must be balanced with an excess in Bi stoichiometry. The elemental composition of the nanocrystals remains unaffected by the thermal annealing, further underscoring that dissolution and growth are unlikely, as this would lead to changes in the elemental composition.

**Table 1:** Atomic Ratios Obtained from the XPS Measurements for AgSbS_2_ Nanocrystals at Different Annealing Times

	RT	30 s	60 s	90 s	120 s
Ag:Sb	1.4	1.4	1.4	1.4	1.2
Ag:S	0.6	0.6	0.6	0.6	0.6
S:Sb	2.2	2.2	2.2	2.2	2.1
Ag:Sb:S	0.6:0.5:1	0.6:0.5:1	0.6:0.5:1	0.6:0.5:1	0.6:0.5:1

Furthermore, no chlorine impurities can be found in the SPLE-treated samples, and the overall amount of carbon is drastically reduced, suggesting that the SPLE treatment with methanol is a very effective process for removing remaining impurities from synthesis from the films and results in well-passivated AgSbS_2_ nanocrystals.

In order to investigate the correlation between the size of AgSbS_2_ nanocrystals and their optical bandgap, the absorbance of MPA-treated AgSbS_2_ films was measured, and the Tauc plot method was employed (see Figure S15). Moreover, ultraviolet photoemission spectroscopy (UPS) was utilized to identify the valence band edge and the Fermi level of the nanocrystals on SnO_2_. As depicted in Figure 7, the bandgap decreases with increasing quantum dot size and improvements in the crystalline order, ranging from 2.0 eV for smaller dots to 1.8 eV for larger ones, which is broader than the value for bulk material of 1.7 eV reported in the literature. This change in bandgap can be attributed to two effects: First, the improvement of crystalline ordering could result in a decrease in bandgap, as has been observed for similar materials.^57^ Second, the lowering of bandgap with increasing NC size could result from changes in quantum confinement. While the exciton Bohr Radius of AgSbS_2_ is unknown and has not been reported in literature so far, exciton Bohr radii for similar compositions have been reported to be in the range of just a few nanometers (see Table S3).^58–61^ Due to the clear and monotonous change in bandgap with nanocrystal size, we believe that changes in quantum confinement are the dominant effect.^54^ As evidenced by our UPS measurements, the valence band of the AgSbS_2_ nanocrystals shifts to lower values with increasing nanocrystal size. These values approach the ones reported by Zhang et al. for bulk AgSbS_2_ (valence and conduction band positions –5.39 eV and −3.39 eV,^62^ respectively). It is possible that the increase in crystalline order, modification of the band structure, as well as the changes in the amount of surface ligands, might be the origin of the lowered valence band positions, however, theoretical modeling would be required to definitively identify the origin of this phenomenon. Notably, the Fermi level of AgSbS_2_ nanocrystals on SnO_2_ after SPLE with MPA differs significantly from that of AgSbS_2_ without ligand exchange (Figure S16). Following passivation with 3-MPA, the Fermi level shifts significantly downward (Figure 7), rendering the material more p-type, while in the case of untreated samples, the material appears intrinsic. Interestingly, after ligand exchange with MPA, the parent bismuth derivate AgBiS_2_ exhibits a strong n-type character.^48,63^ Consequently, MPA passivation not only minimizes surface defects and trap states, thereby enhancing the stability of NCs but also alters the energetic properties of the material.

To explore the photovoltaic performance of AgSbS_2_ nanocrystals, we incorporated the most crystalline (size of approximately 9 nm) nanocrystals with MPA-ligands in planar solar cells and compared their performance to the as-synthesized AgSbS_2_ nanocrystals with MPA but without thermal treatment in solution. We adopted the n-i-p architecture, commonly used for the related AgBiS_2_ nanocrystals, as illustrated in Figure 7b.^48,57^ Similarly to the photoemission measurements, AgSbS_2_ nanocrystal layers were deposited on a ca. 30 nm thin layer of SnO_2_ nanoparticles on ITO. After the deposition of three layers of AgSbS_2_ nanocrystals via the SPLE with MPA, a final absorber layer thickness of approximately 40 nm was achieved. The formed AgSbS_2_ nanocrystal layers are smooth, compact, and dense and free of structural defects such as pinholes or cracks as confirmed by scanning electron microscopy (SEM) (Figure S17). Subsequently, a few nanometer thick poly­[bis­(4-phenyl)-(2,4,6-trimethylphenyl)­amine] (PTAA) layer was deposited on top, and the devices were completed by thermal evaporation of MoO_*x*_ and silver as a non-transparent electrode. A cross-section image of the fabricated solar cells confirming the vertical devices architecture is shown in Figure S18. Figure 7c illustrates a simplified energy level diagram for photovoltaic devices with the largest and most crystalline AgSbS_2_ nanocrystals, while interfacial and charge-transfer effects are not considered. PTAA and SnO_2_ were chosen due to their compatibility with the energetics of the AgSbS_2_ layer and orthogonal processing requirements. Figure 7d shows the current density–voltage (*J*-*V*) characteristics of the corresponding AgSbS_2_-based solar cells after 1 week of shelf-storage. The as-deposited AgSbS_2_-nanocrystals exhibit a low photovoltaic performance with considerable hysteresis. The thermally annealed AgSbS_2_ nanocrystals, on the other hand, exhibit a hysteresis-free photovoltaic performance with a short circuit current density (*J*_sc_) of 10.59 mA/cm^2^, an open circuit voltage *V*_oc_ of 0.45 V, a fill factor (FF) of 42%, and a PCE of 1.99% for the best-performing solar cell (Table S4). Solar cells fabricated from nanocrystal inks with annealing times below 120s exhibited only a small performance improvement, while nanocrystals annealed for 120s show by far the best solar cell performance, probably due to the most suitable crystalline order and bandgap, as well as favorable energetic alignment in the chosen device structure. We note that similar to many other nanocrystal solar cells, the AgSbS_2_ nanocrystal solar cells exhibit their best performance after 6 days of storage in ambient (Figure S19); while the exact origin for this behavior remains unclear in this case, it has been suggested that the aging of the PTAA:MoO_*x*_ layer is responsible for this improvement.^57^ Beyond these proof-of-concept photovoltaic devices, future optimization of the device architecture, ligand chemistry, and modifications of the HTL and ETL might result in more stable and improved performing solar cells. To confirm that the active layer of the fabricated solar cells consists of AgSbS_2_ nanocrystals and not bulk AgSbS_2_, we conducted XRD measurements on the active layer of such solar cells. For this, we removed the HTL and top electrode via scotch tape to gain access to the thin nanocrystal layer. Despite the very thin active layer of just a few dozen nanometers, grazing incident measurements with high power and point focus allowed us to probe the diffraction pattern of the device (Figures S20 and S21). While the diffractograms are dominated by the highly crystalline SnO_2_ layer, the corresponding broad reflections of AgSbS_2_ nanocrystals can be observed only in the case of layers fabricated from annealed AgSbS_2_ at 120 °C in solution. The absence of sharp, intense reflections confirms that the layer deposition, ligand exchange and annealing of the film do not result in bulk AgSbS_2_ layers, thus preserving their nanocrystal nature.

**Figure 7:**
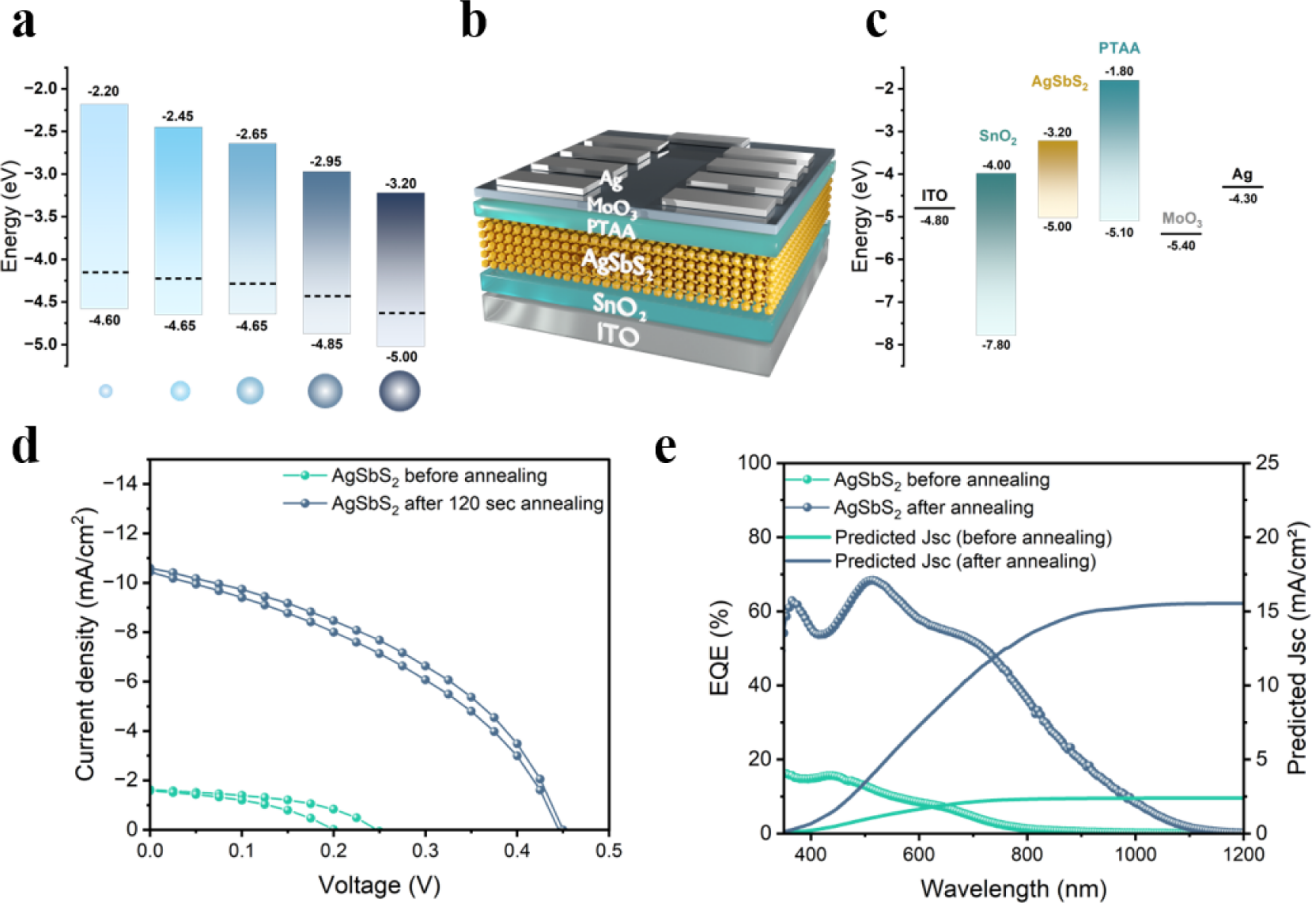
(a) UPS data of AgSbS_2_ nanocrystal films on SnO_2_ after different annealing times in solution. (b) schematic device architecture of planar AgSbS_2_ solar cells. (c) schematic energy level diagram for such AgSbS_2_ solar cells. (d) best current-density–voltage characteristics for AgSbS_2_ nanocrystal solar cells, and e) external quantum efficiency spectra of solar cells before and after annealing.

The external quantum efficiency (EQE) spectra demonstrate a similar shape for both types of solar cells, exhibiting a substantial enhancement from 15% to 55% at 400 nm and from 10% to 60% at 600 nm, as well as an apparent redshift of the entire spectrum, thereby confirming the narrowing of the band gap and the increasing size of the nanoparticles (Figure 7e,d). We note that the red-shift goes beyond what is expected from the bandgap narrowing and is likely associated with the formation of absorbing sub-bandgap states (Figure S15). The predicted current densities *J*_sc_ from the EQE spectra differ substantially from those recorded under one sun illumination. This discrepancy might originate from the fact that these measurements are performed under substantially different light intensities, thus suggesting a nonideal behavior at higher light-intensity, such as those for *J*-*V* characterization. The reduction in the measured *J*_sc_ may originate from a high trap density within the nanocrystal layer, however, a more detailed investigation of this observation is beyond the scope of this work. Nevertheless, to the best of our knowledge, the reported PCEs for the annealed AgSbS_2_ NC are among the highest reported values for AgSbS_2_ solar cells.

The performance reached in our study and in previous works exploring this material in its bulk form lag behind those demonstrated for other materials such as AgBiS_2_, which reach PCE above 10%. Despite this, AgSbS_2_ has the potential to reach far higher performance upon further optimization of the synthesis and device structure, with a theoretical maximum of over 30%. The advantage of this system lies in its favorable bandgap of ∼1.7 eV, which is desirable for a potential application in tandem photovoltaics with silicon bottom cells.

## Experimental Section

### Materials

Silver nitrate (AgNO_3_, 99.5%) was purchased from Grüssing GmbH. Antimony chloride (SbCl_3_, ≥99.95%), oleylamine (OAm, 98%), trioctylphosphine (TOP, 90%), 3-mercaptopropionic acid (MPA, 98%), sulfur powder (S, 99.98%) and lithium aluminum hydride powder (LiAlH_4_, 95%), and deionized water were purchased from Sigma-Aldrich. Anhydrous toluene (99.85%), methanol (99.8%), and acetone (99.8%) were purchased from Thermo Scientific. Tetrahydrofuran (THF, 99.8%), diethyl ether (99.5%), sodium chloride (NaCl, 99.5%), and sodium sulfate (Na_2_SO_4_, 99%, anhydrous) were purchased from Fisher Chemical. Lauroyl chloride (La_2_Cl, 98%) was purchased from TCI. Tin oxide (SnO_2_, 15% in H_2_O) was purchased from Alfa Aesar. Poly­[bis­(4-phenyl)-(2,4,6-trimethylphenyl)­amine] (PTAA, Mw 30,000) was purchased from Ossila. Molybdenum oxide (MoO_3_, 99.95%) and silver pellets (Ag, 99.99%) were purchased from Kurt J. Lesker. ITO substrates were purchased from Yingkou Shangneng Photoelectric Material Co.

### La_2_S Synthesis

La_2_S was synthesized using a synthetic approach published by Koketsu et al.^64^ S powder (1360 mg, 42.5 mmol) was dissolved in 300 mL of THF at room temperature and under a nitrogen atmosphere. Then, LiAlH_4_ (1344 mg, 35.4 mmol) as a suspension in 50 mL of THF was added to the flask in several portions under stirring (Note: gas formation occurs during the mixing of LiAlH_4_ with THF and its addition to the reaction system). The mixture was stirred for 40 min, resulting in a gray LiAlHSH suspension. Then, lauroyl chloride (24.6 mL, 106 mmol) was added dropwise into the LiAlHSH suspension under vigorous stirring. The reaction mixture was stirred at room temperature for 3 h under a nitrogen atmosphere. Then, 10 mL of deionized water was added to quench any unreacted chloride. The mixture was extracted with diethyl ether (450 mL) and washed six times with saturated NaCl solution. The organic layer was dried over sodium sulfate, filtered, and evaporated to dryness. The resulting colorless powder was recrystallized twice using diethyl ether and dried under vacuum overnight.

### Ag_2_S Synthesis

Ag_2_S NCs were synthesized according to a previously reported procedure.^48^ The silver precursor was obtained by dissolving 102 mg (0.6 mmol) of AgNO_3_ in 4 mL of toluene and 3 mL of oleylamine. 120 mg (0.3 mmol) of La_2_S was dissolved in 1 mL of toluene and both precursors were stirred separately for 1 h. The sulfur precursor was then rapidly injected at room temperature into the silver precursor solution, and the system was further stirred for 2 h. After the reaction, the NCs were precipitated with methanol and centrifuged at 6000 rpm for 5 min. Finally, the Ag_2_S NCs were redispersed in dry toluene and filtered with a PTFE filter (pore size: 0.22 μm).

### Cation Exchange

For the cation exchange reaction, Ag_2_S NCs dispersed in dry toluene were used with a concentration of 10 mg/mL. Then 6.84 mg (0.03 mmol) of SbCl_3_ in 70 μL of toluene and 30 μL of TOP were quickly introduced into the Ag_2_S NCs and stirred at room temperature for 5 min. After the reaction, which resulted in a rapid color change from dark brown to orange, AgSbS_2_ was purified once with acetone and centrifuged at 6000 rpm for 5 min. The purified AgSbS_2_ NCs were redispersed in dry toluene and filtered with a PTFE filter (pore size: 0.22 μm). For postsynthetic size-tuning, the NC dispersion was placed on a preheated hot plate in a heated aluminum block at 150 °C in a small vial and kept there under stirring for the reported amount of time.

### Device Fabrication

ITO substrates were preliminarily cleaned with acetone and isopropanol in an ultrasonication bath for 30 min. After that, substrates were additionally cleaned with oxygen plasma (0.4 mbar) for 10 min. SnO_2_ as electron transporting layer was prepared by diluting the commercially available solution in a ratio of 1:2 (v/v) with deionized water. The tin oxide solution was then statically spin-coated at 2000 rpm for 30 s and subsequently annealed at 270 °C for 15 min. After that, AgSbS_2_ NCs solution with a concentration of 20 mg/mL in toluene was spin-coated at 2000 rpm, treated statically with 3-MPA (1% v/v in dry methanol) for 45 s, followed by washing two times with methanol and once with toluene to remove residual impurities. This procedure was repeated three times. After that, the films were annealed at 150 °C for 30 s inside a nitrogen-filled glovebox. Prepared PTAA solution (1.6 mg/mL in toluene) was statically spin-coated at 2000 rpm for 30 s onto the AgSbS_2_ layer. Finally, 3 nm of MoO_3_ and 120 nm of Ag were thermally evaporated through a shadow mask with a final pixel area of 4.5 mm^2^.

### UV–Vis

A Jasco V-770 spectrometer was used to collect the absorption spectra in the ultraviolet–visible and near-infrared regions. Absorbance measurements were carried out in 1 cm wide quartz cuvettes.

### Time-Resolved Absorbance

For the time-resolved absorbance measurements, the NC solution at a concentration of 10 mg/mL in toluene was filled into a quartz cuvette with an optical path length of 10 mm. The cell was placed in an aluminum cuvette holder, which was equipped with a light source (Thorlabs SLS201L) on one side and an optical fiber output on the other side. The cuvette holder was placed on a hot plate at 150 °C. After preheating the entire setup, the cuvette containing the NC solution was placed in the holder. Optical density was recorded at the fiber output using a compact spectrometer (Thorlabs CCS200/M). Absorbance measurements were taken every 10 s for a total amount of 1200 s.

### X-ray Diffraction

Diffraction measurements were carried out on a Bruker D8 Discover diffractometer, with a Cu anode (K_α_ λ = 1.5406 Å) in a coupled Θ/2Θ scan with a 1D-detector. Samples were prepared by drop-casting the nanoparticle solution onto a glass substrate with an area of 1 cm^2^ without performing any ligand exchange procedures.

For the active layers of solar cells, grazing incidence measurements with a collimated beam (0.2 mm) at an incidence angle of 0.4° have been recorded on a Rigaku Smart Lab diffractometer equipped with a 2D HyPix3000 detector and a rotating copper anode at 8.1 kW. Prior to measurements, the active layer of the solar cell has been exposed by applying scotch tape to the silver electrode and HTL-side of the solar cell, followed by a quick removal of the scotch tape leaving no visual residual of the electrode or HTL. 1D diffraction patterns were obtained after integrating the 2D diffraction map and a background correction utilizing the Rigaku Smart Lab Studio II software.

### Transmission Electron Microscopy

A Jeol JEM F200 with an acceleration voltage of 200 kV was used for transmission electron microscopy (TEM) at the Dresden Center for Nanoanalysis (DCN). Carbon grids were used as substrates for drop-cast NCs solutions, heavily diluted in toluene. The average QD size was calculated by measuring the diameters of at least 100 nanoparticles.

### Scanning Electron Microscopy

SEM imaging was performed on a Analytical SEM Zeiss Gemini 500 at a chamber pressure below 10^–6^ mbar. Samples were mounted on standard SEM holders using conductive silver paste to avoid sample charging. Images were collected at an acceleration voltage of 1.5 kV using the In-lense and ESB detector of the Zeiss Gemini SEM. For cross-section images, samples were scratched with a diamond pen at the backside of the substrate and broken into half with mechanical force. Prior to imaging, a 2 nm carbon coating was applied to lower sample charging.

### X-ray Photoemission Spectroscopy and Ultraviolet Photoelectron Spectroscopy

X-ray photoemission spectroscopy (XPS) and Ultraviolet photoelectron spectroscopy (UPS) were performed on AgSbS_2_ NCs deposited via spin-coating on SnO_2_/ITO substrates and annealed at 150 °C for 30 s with and without performing ligand exchange procedures. XPS measurements were carried out on an ESCALAB 250Xi by Thermo Scientific in an ultrahigh vacuum chamber (base pressure: 2 × 10^–10^ mbar) with an XR6 monochromated Al Kα X-ray source (hν = 1486.6 eV) and a pass energy of 20 eV. The C 1s state (284.8 eV) was used as a reference to calibrate the binding energy for all presented XPS spectra due to minute differences in surface charging. UPS measurements were conducted with double differentially pumped He discharge lamp (hν = 21.22 eV) with a pass energy of 2 eV and a bias of −10 V.

### Solar Cell Characterization

A Keithley 2450 source measure unit and an Abet A+++ solar simulator with an AM 1.5 filter were used for current–voltage measurements. Solar cells were measured each day, starting at day 0, directly after fabrication. During the measurements, the samples were stored in ambient conditions (20 °C < *T* < 23 °C; 30% < R.H. < 65%) in a cabinet. The performance increases within the first few days, reaches a maximum after 6 days, and remains roughly stable (Figure S19). This behavior is common for quantum dot-based solar cells.

### Nuclear Magnetic Resonance Spectroscopy (NMR)

NMR data were recorded at ambient temperature on a Bruker Avance III 600 spectrometer equipped with a BBI probe operating at 600.2 MHz for ^1^H and 150.9 MHz for ^13^C. Chemical shifts δ are given in ppm relative to TMS. The solvent signals were used as reference (CDCl_3_: δ_Η_ 7.260 ppm residual CHCl_3_, δ_C_ 77.16 ppm, Tol-*d*_8_ δ_Η_ 2.080 ppm residual CHD_2_-C_6_D_5_). Coupling constants *J* are given in Hertz and were determined assuming first-order spin–spin coupling. DOSY measurements were performed using the standard Bruker pulse sequence ledbpgp2s.

### Fourier Transform Infrared Spectroscopy (FTIR)

FTIR measurements was performed using a JASCO FT/IR-4XLE spectrometer with a wavenumber range of 4000–1000 cm^–1^, a resolution of 4 cm^–1^, and a scan speed of 2 mm/s. A high-intensity ceramic was used as the light source with a DLATGS detector. Measurements were conducted as powder after complete solvent evaporation on an ATR PRO 4X unit with a PKS-D1 diamond at an incident angle of 45° utilizing one reflection.

## Conclusion

This study demonstrated a cation exchange synthesis of AgSbS_2_ nanocrystals at room temperature and ambient conditions. For this, we utilized Ag_2_S nanocrystals of ca. 4 nm size obtained by bis­(lauroyl) sulfide, followed by cation exchange with SbCl_3_ in the presence of TOP. We show that the reaction conditions result in a fast and complete exchange of Ag^+^ with Sb^3+^ and the formation of AgSbS_2_ nanocrystals with ideal stoichiometry. The cation exchange reaction also results in an exchange of surface ligands on the AgSbS_2_ nanocrystals, enabling a unique and fast postsynthetic size-tunability in solution while maintaining the overall nanocrystal composition. We show that 3-MPA can act as a short-chained ligand and enables a layer-by-layer deposition of AgSbS_2_ nanocrystal films for optoelectronic applications. The resulting nanocrystal films are p-type and act as absorber layers in planar nanocrystal solar cells. Such photovoltaic devices exhibit a maximum power conversion efficiency (PCE) of up to 1.99%. These initial results underline the great potential of the ternary AgSbS_2_ material. With its p-type character and wider bandgap compared to the parent AgBiS_2_, these nanocrystals are a potential candidate for future tandem and or multijunction solar cells.

## Supplementary Material



## Notes

NMR and FTIR data for La_2_S and nanocrystals; TEM images; size distribution histograms; photographs of colloidal nanocrystal solutions; XPS and UPS spectra; tables for elemental compositions; SEM images of films and devices; 2D-XRD and device stability data (PDF)
