# Supplementary material for: Low-Temperature Synthesis and Postsynthetic Size-Tunability of AgSbS2 Nanocrystals and Their Application in Planar Solar Cells
Source: ACS Appl Energy Mater. 2026 Jan 5;9(4):2067–79. doi: 10.1021/acsaem.5c03034 (PMC12933516; doi:10.1021/acsaem.5c03034)
Supplement: Supplementary file 1 [file ae5c03034_si_001.pdf]

SUPPORTING INFORMATION

Low temperature synthesis and post-synthetic size-tunability of $AgSbS_2$ nanocrystals and their application in planar solar cells

Alina Senina,[1] Anatol Prudnikau,[1] Angelika Wrzesińska-Lashkova,[1,2] Julius Brunner,[1,2] Xuan Qi,[1] Vladimir V. Shilovskikh,[1,2] Yana Vaynzof,[1,2] Tilo Lübken,[3] Fabian Paulus[1,4,]*

[1] Leibniz Institute for Solid State and Materials Research (IFW) Dresden, Helmholtzstraße 20, 01069 Dresden, Germany

[2] Chair for Emerging Electronic Technologies, Technical University of Dresden, Nöthnitzer Str. 61, 01187 Dresden, Germany

[3] Chair of Organic Chemistry I, Technische Universität Dresden, Bergstraße 66, 01069 Dresden, Germany

[4] Center for Advancing Electronics Dresden (cfaed), Technische Universität Dresden, Helmholtzstraße 10, 01069 Dresden, Germany

* corresponding author: f.paulus@ifw-dresden.de

^{1}H NMR (600 MHz, chloroform-d) δ [ppm] = 2.73 (t, J=7.4, 4H), 1.65 (p, J=7.4, 4H), 1.36 – 1.20 (m, 32H), 0.88 (t, J=7.0, 6H).

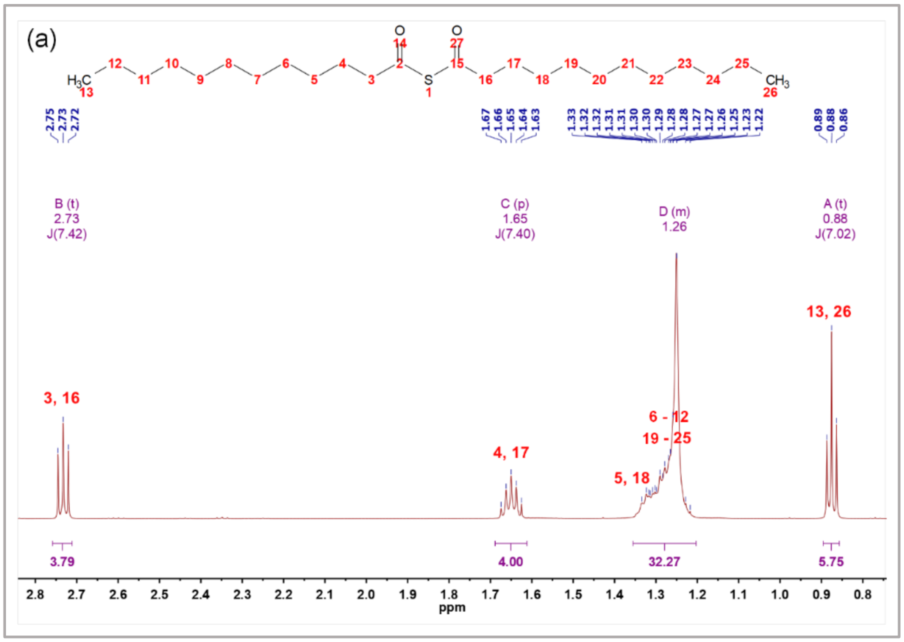

^{13}C NMR (151 MHz, Chloroform-d) δ [ppm] = 195.19, 46.18, 32.04, 29.73, 29.70, 29.52, 29.47, 29.36, 28.95, 24.86, 22.83, 14.27.

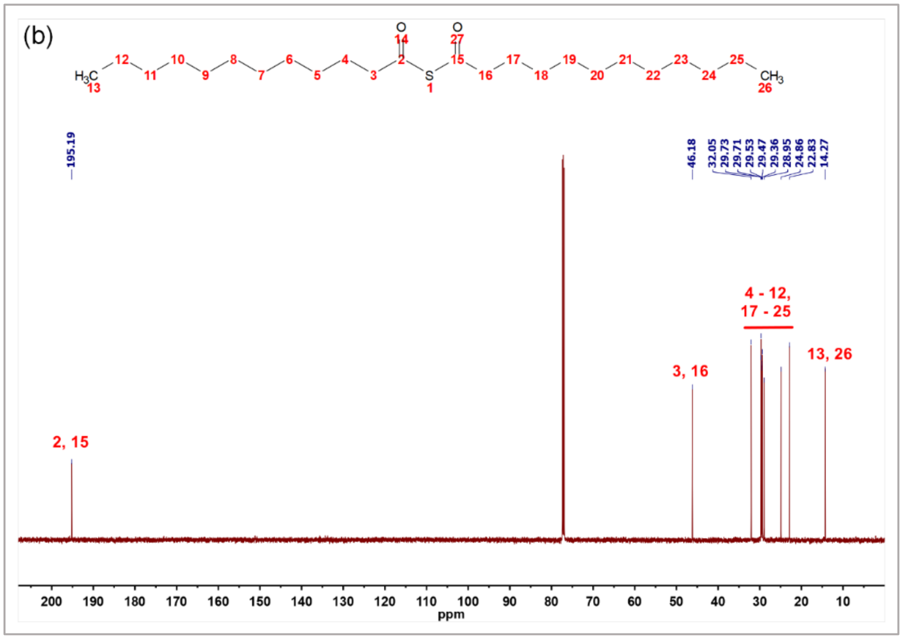

Figure S1. ^{1}H NMR (a) and ^{13}C (b) NMR spectra of bis(lauroyl) sulfide (chloroform-d).

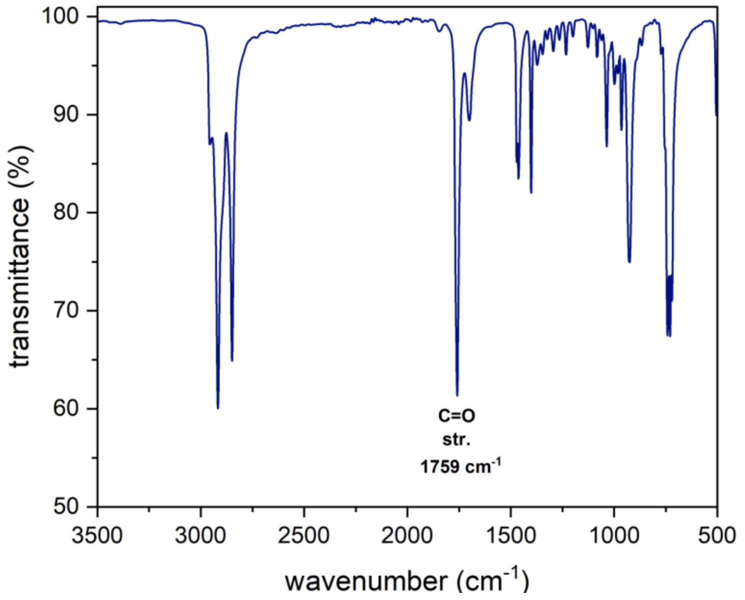

Figure S2. ATR FTIR spectrum of bis(lauroyl) sulfide powder.

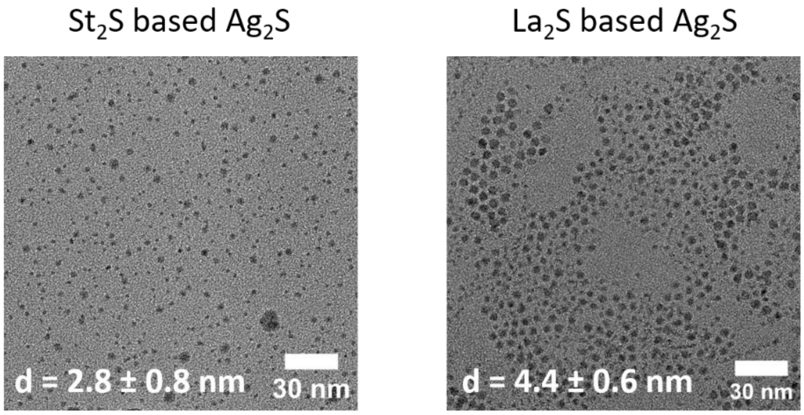

Figure S3. TEM images of Ag_2S NCs synthesized with bis(stearoyl) sulfide and bis(lauroyl) sulfide.

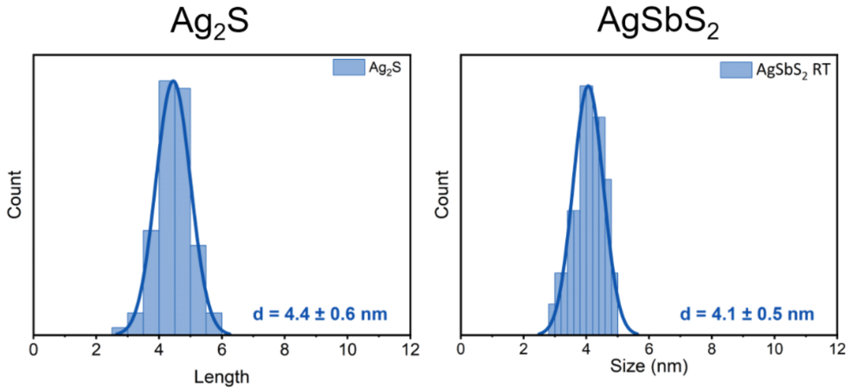

Figure S4. Size distribution of Ag_2S and $AgSbS_2$ NCs after cation exchange.

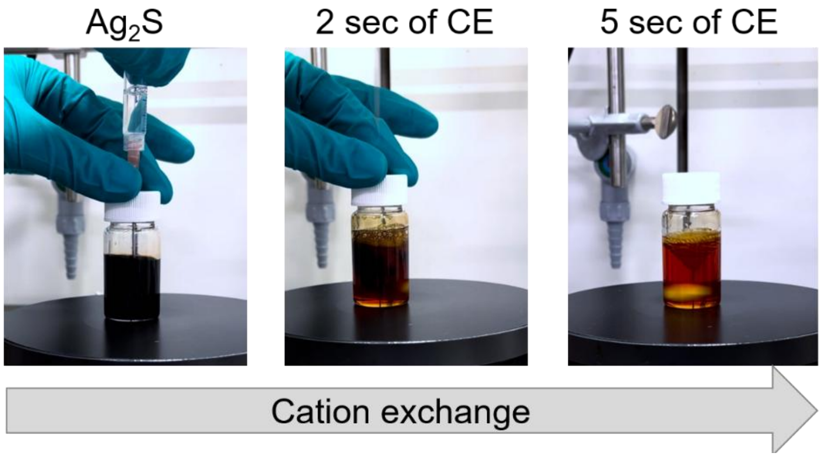

Figure S5. Change of the color in the first seconds of the CE reaction.

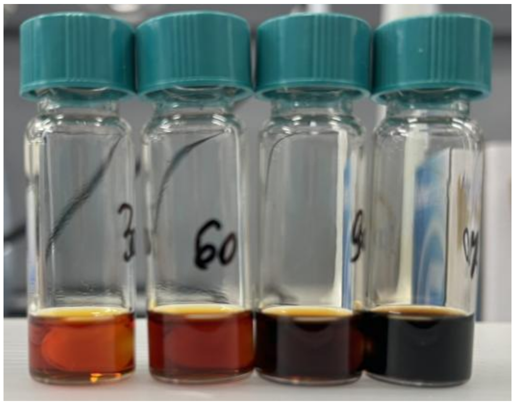

Figure S6. Color change of $AgSbS_2$ nanocrystal dispersion upon thermal annealing for 30 s (left), 60 s (second from the left), 90 s (third from left) and 120 s (right). The corresponding UV-VIS absorbance spectra are shown in Figure 2a.

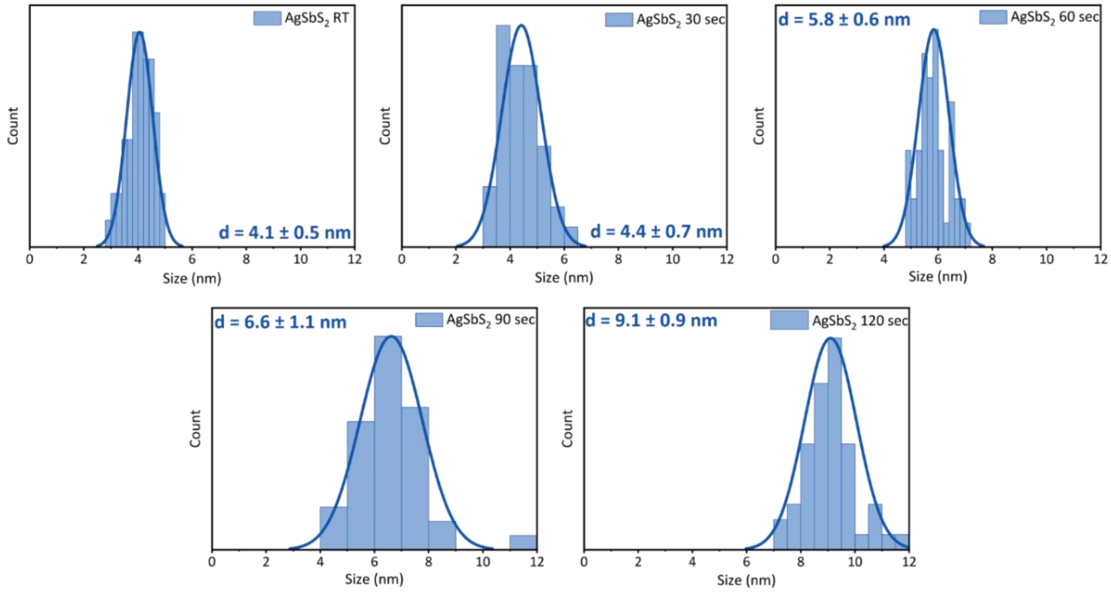

Figure S7. Size distribution of $AgSbS_2$ nanocrystals from Figure 2c throughout the thermal annealing in solution.

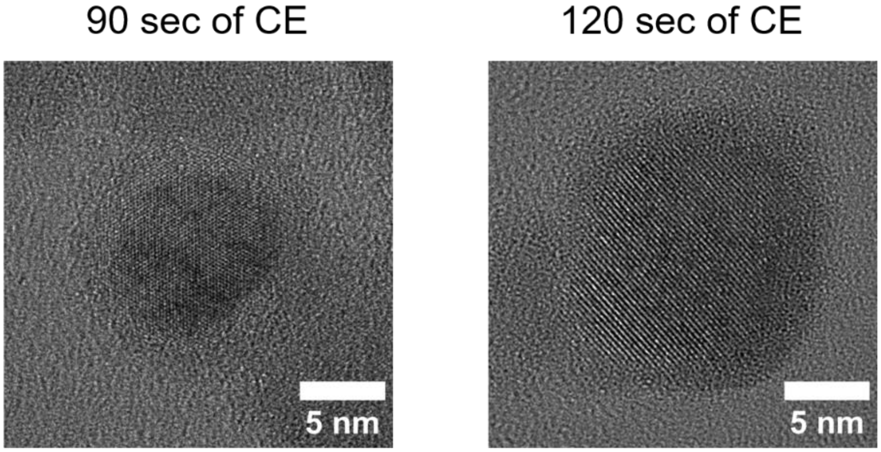

Figure S8. TEM images of $AgSbS_2$ quantum dots after 90 and 120 seconds of annealing confirm an improvement in crystallinity and the appearance of lattice fringes in high-resolution images.

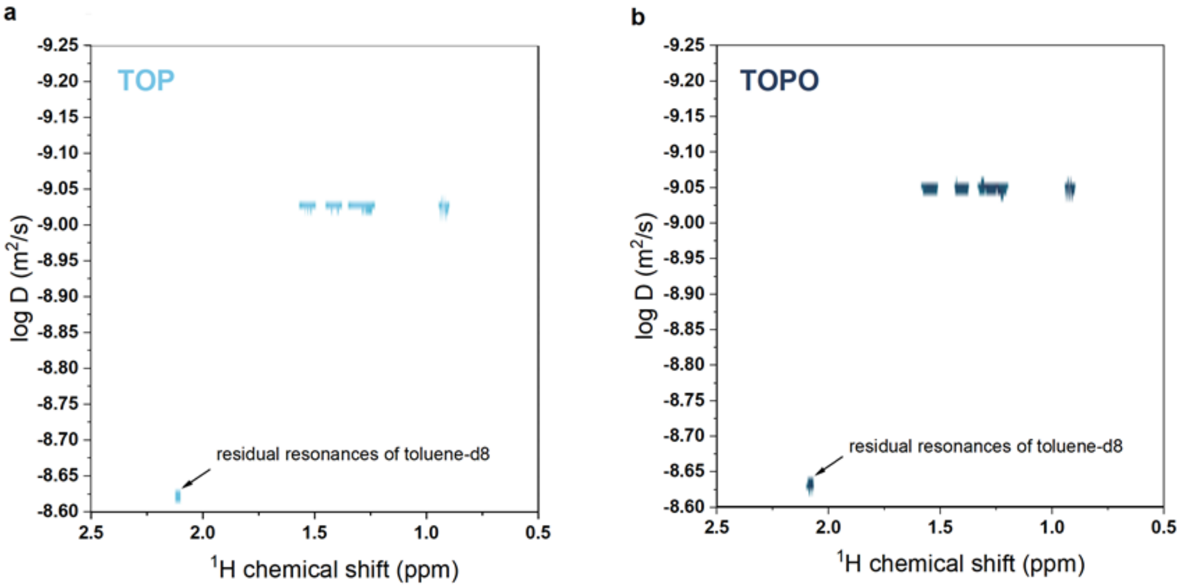

Figure S9. DOSY NMR spectra of TOP (a) and TOPO (b) in toluene-d8.

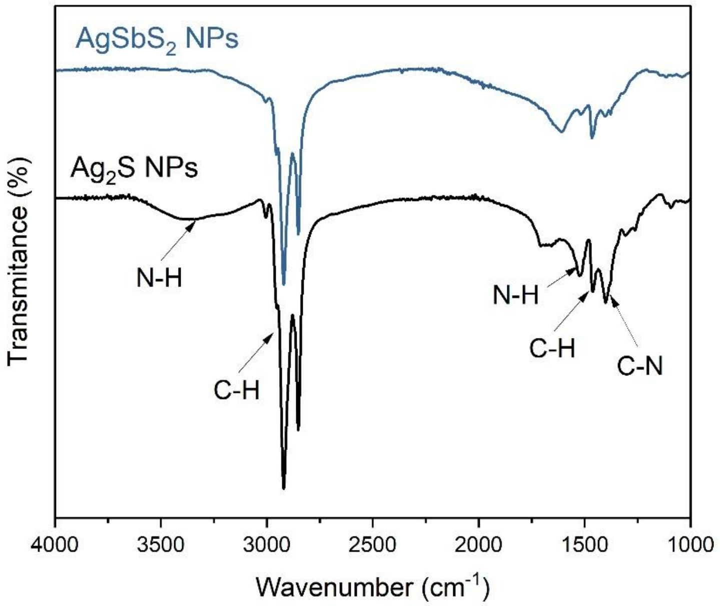

Figure S10. FTIR spectra of Ag_2S and $AgSbS_2$ nanoparticles, revealing the change in ligand chemistry before and after cation exchange reaction.

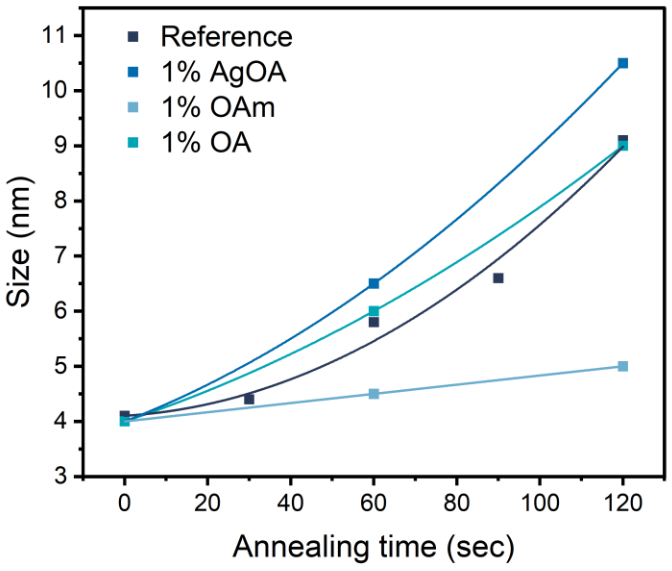

Figure S11. Temporal evolution of the NC sizes of the NC shown in Figure 5 during thermal annealing in the presence of different types of ligands. The 'reference' refers to NCs annealed in solution as shown in figure 2.

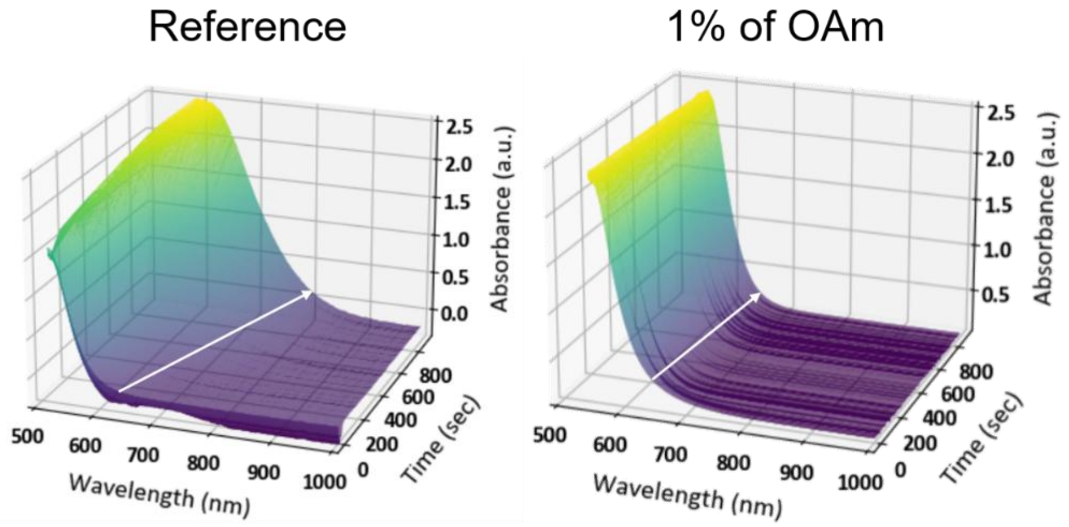

Figure S12. Absorbance surface of $AgSbS_2$ QDs without and with the addition of 1% of OAm. The 'reference' refers to NCs annealed in solution as shown in figure 2.

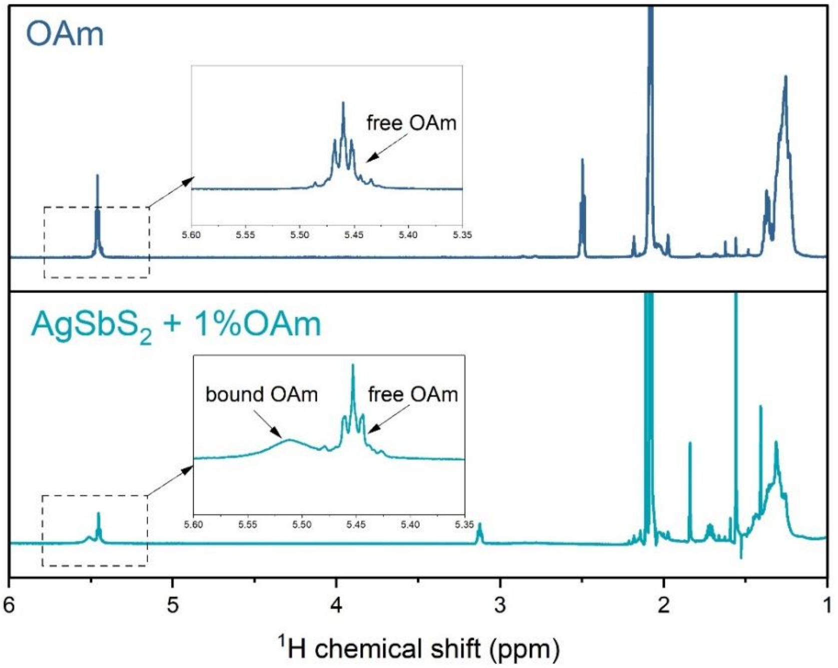

Figure 13: NMR spectra of plain OAm (top) and $AgSbS_2$ nanocrystals (bottom) treated with 1% OAm, annealed (similar to Figure 5, S11, and S12), and purified to remove excess OAm.

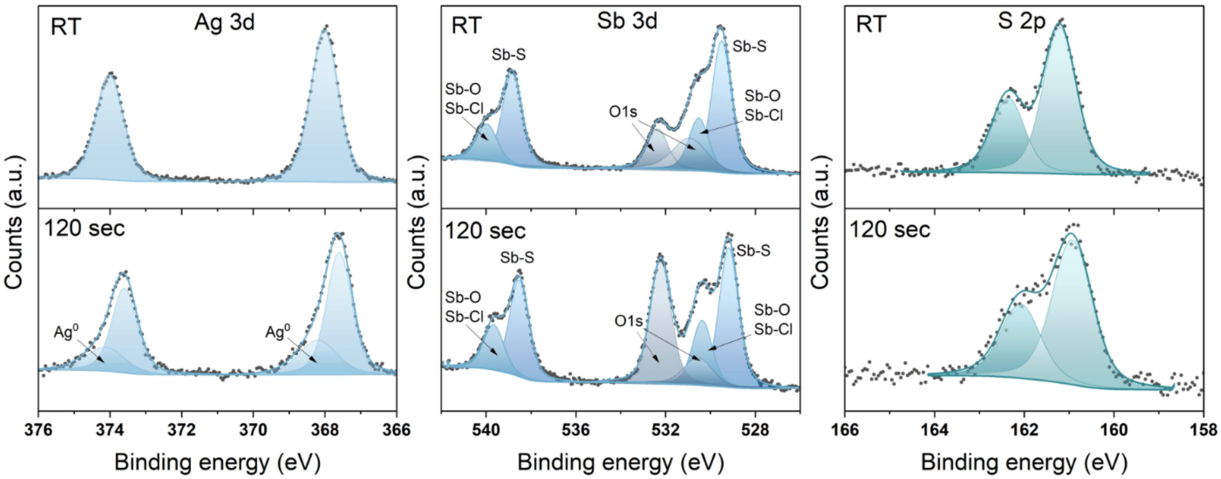

Figure S14. XPS spectra of Ag 3d, Sb 3d, and S 2p for spin-coated $AgSbS_2$-nanocrystals on SnO_2 directly after synthesis and after annealing for 120°C in solution.

Table S1. Atomic percentages and atomic ratios obtained by XPS measurement on $AgSbS_2$-nanocrystals spin-coated on SnO_2 **without any further treatment**.

	**RT**	**120 sec**
**Ag**	2.43	1.18
**Sb**	3.25	1.48
**S**	4.85	2.94
**C**	72.90	81.65
**O**	13.68	10.04
**P**	1.32	0.43
**Cl**	1.56	1.02
**Ag : Sb**	0.75	0.79
**Ag : S**	0.5	0.4
**S : Sb**	1.5	1.99
**Ag : Sb : S**	0.5 : 0.7 : 1	0.4 : 0.5 : 1

Table S2. Atomic percentages obtained by XPS measurement on $AgSbS_2$-nanocrystals films after different annealing-times spin-coated on SnO_2 and **SPLE-treatment with MPA** in methanol.

	**RT**	**30sec**	**60 sec**	**90 sec**	**120 sec**
**Ag**	19.21	20.12	19.80	16.29	18.05
**Sb**	13.39	14.20	14.10	11.51	14.08
**S**	29.80	31.45	30.88	25.46	31.65
**C**	27.09	26.53	24.89	18.91	15.93
**O**	8.44	6.59	8.69	21.55	14.56
**P**	2.08	1.11	1.64	0.86	1.14
**Cl**	-	-	-	-	-
**Sn**	-	-	-	5.42	3.87

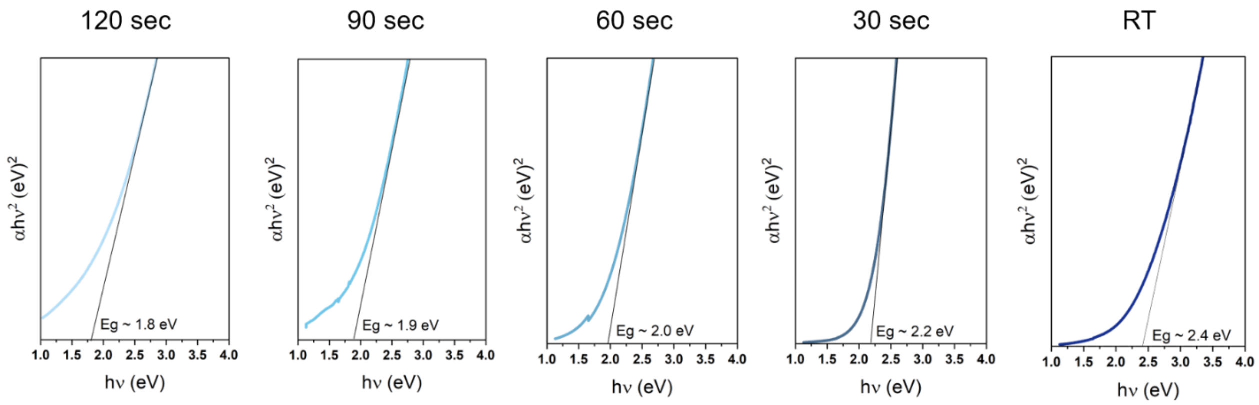

Figure S15. Tauc plots of $AgSbS_2$ films at different annealing times. Average film thicknesses for 120 s, 90 s, 60 s, 30 s and RT were 28 nm, 22 nm, 27 nm, 25 nm, and 21 nm, respectively.

Table S3. Comparison of Exciton Bohr-Radii for other I-III-VI materials.

**Material System**	**Reported Exciton Bohr Radius**	**Reference**
$AgBiS_2$	4.6 nm	F. Viñes, M. Bernechea, G. Konstantatos and F. Illas, *Phys. Rev. B*, 2016, **235203**, 1–8 https://doi.org/10.1103/PhysRevB.94.235203
$AgInS_2$	5.5 nm	Y. Hamanaka, T. Ogawa, M. Tsuzuki and T. Kuzuya, *J. Phys. Chem. C*, 2011, **115**, 1786–1792 https://doi.org/10.1021/jp110409q
$CuInS_2$	3.8 nm	M. V. Yakushev, R. W. Martin, A. V. Mudryi and A. V. Ivaniukovich, *Appl. Phys. Lett.*, 2008, **92**, 2–5. https://doi.org/10.1063/1.2896301
$CuGaS_2$	4.0 nm	L. Yang, S. Zhang, B. Xu, J. Jiang, B. Cai, X. Lv, Y. Zou, Z. Fan, H. Yang and H. Zeng, *Nano Lett.*, 2023, **23**, 2443–2453 https://doi.org/10.1021/acs.nanolett.2c03138

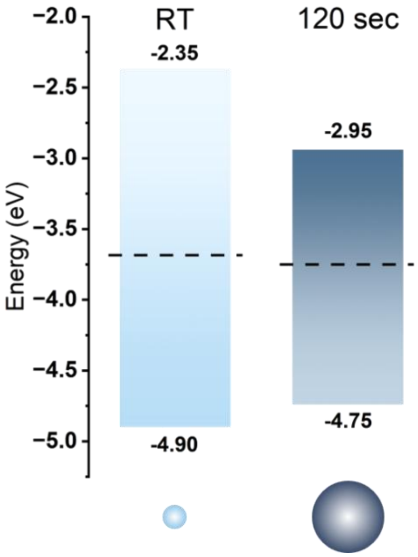

Figure S16. UPS data of $AgSbS_2$ QDs without 3-MPA treatment.

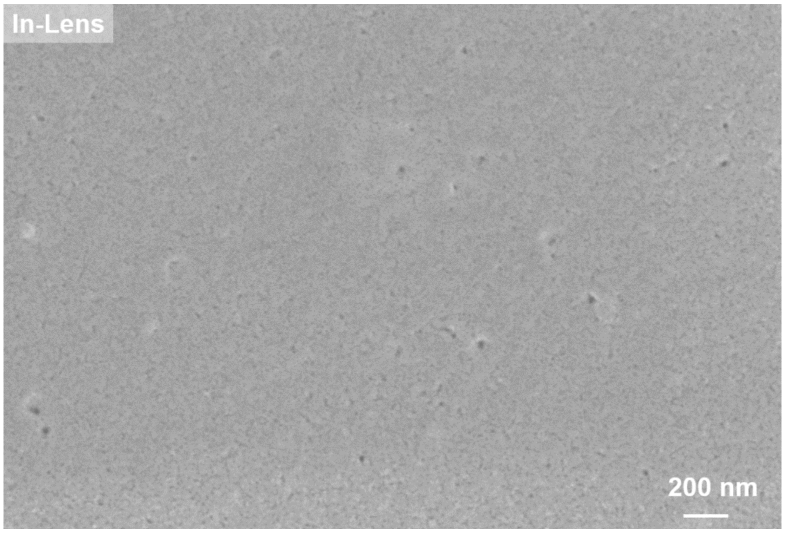

Figure S17. SEM surface view of a $AgSbS_2$ nanocrystal film after ligand exchange with MPA.

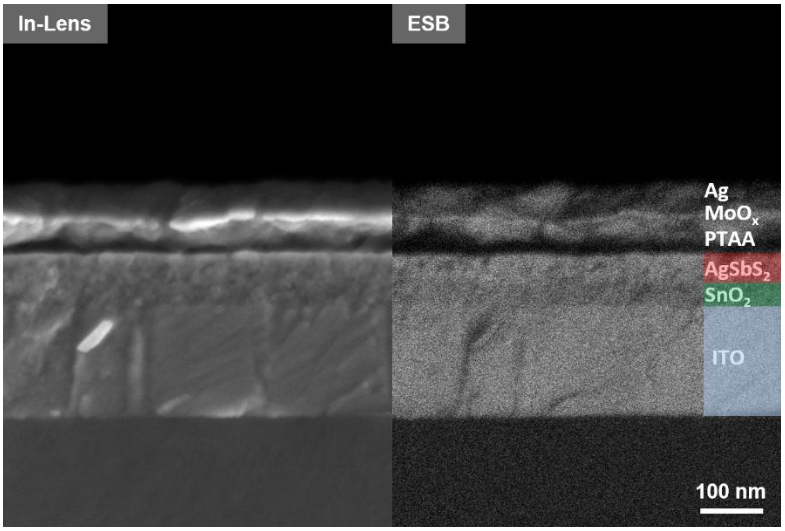

Figure S18. Cross-section SEM image of the a $AgSbS_2$ solar cell. Left: region images with the Zeiss Gemini In-Lense detector, right: same region visualized with the electron Zeiss Gemini ESB detector with improved material contrast. The delamination of the PTAA layer and top electrode from the nanocrystal film is the results from mechanical breaking the functional device and cannot be avoided. In the working solar cells, the layers are in ideal contact.

Table S4. Photovoltaic parameters and standard deviation of $AgSbS_2$ solar cells before and after thermal annealing in solution.

	Voc (V)	Jsc (mA/cm^2)	FF (%)	PCE (%)
$AgSbS_2$ after annealing,in solution **120s, 150°C**	0.46±0.03	-5.98±2.96	0.42±0.05	1.14±0.52
Champion device	0.45	-10.59	0.42	1.99
$AgSbS_2$ **before annealing**	0.26±0.04	-1.57±0.11	0.43±0.04	0.14±0.02
Champion device	0.25	-1.63	0.46	0.18

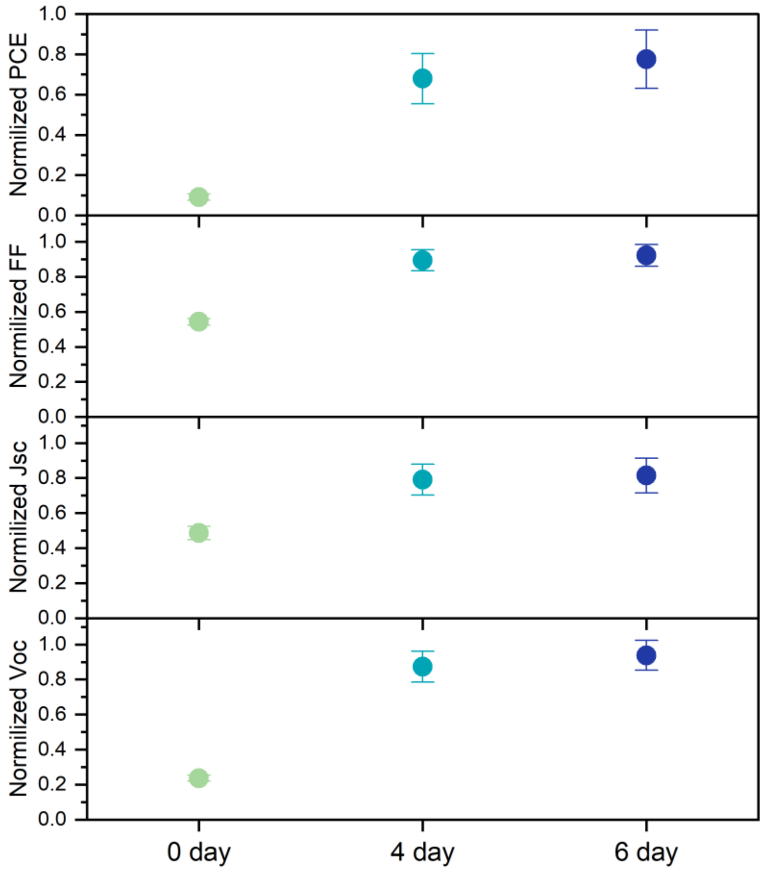

Figure S19. Evolution of the solar cell parameters and performance upon storage in ambient and electrical measurements on days 0, 4, and 6. Normalized to the best (highest) value obtained. For solar cells, fabricated with nanocrystals annealed 120s.

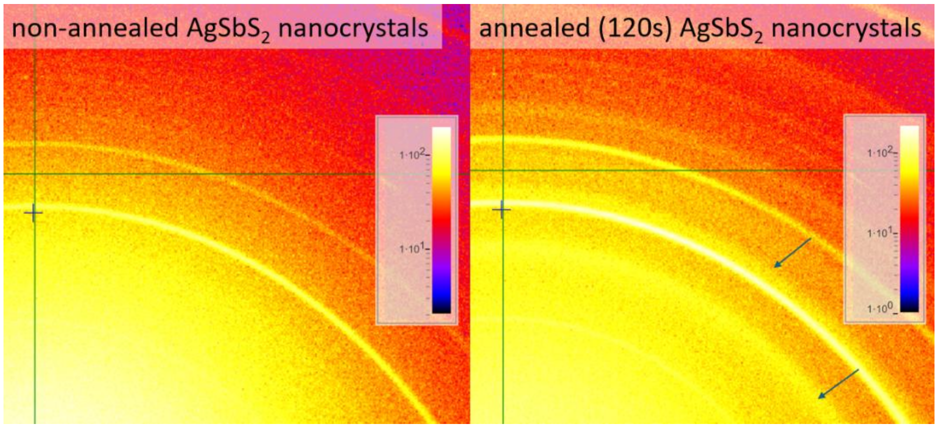

Figure S20. Gracing Incidence 2D-XRD on the active layer of $AgSbS_2$ solar cells with (left) $AgSbS_2$ nanocrystals (RT, no annealing in solution) and $AgSbS_2$ annealed nanocrystals (120s, 150°C). The arrows indicate the presence of broad diffraction rings in the case of the annealed nanocrystals.

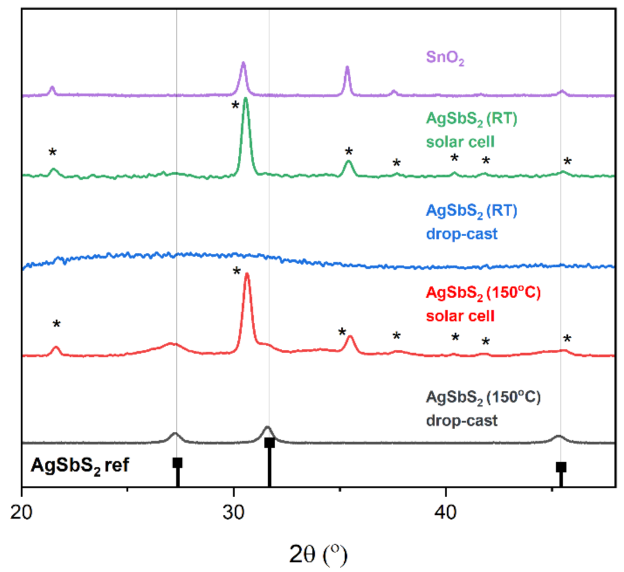

Figure S21. 1-dimensional diffraction profiles obtained after integration of the diffraction maps shown in Figure S18 for the active layer of solar cells for annealed (red) and non-annealed nanocrystals (green). Included for comparison are diffraction pattern of a plain SnO_2 ETL without AgSbS2 nanocrystals (purple) and the corresponding XRD data from the drop-casted nanocrystal dispersion shown in Figure 2 for the nonannealed (blue) and annealed (black) $AgSbS_2$ nanocrystals.
